# Supplementary material for: Probing optical anapoles with fast electron beams
Source: Nat Commun. 2023 Dec 20;14:8478. doi: 10.1038/s41467-023-43813-y (PMC10733292; doi:10.1038/s41467-023-43813-y)
Supplement: Supplementary file 1 — Supplementary Information [file 41467_2023_43813_MOESM1_ESM.pdf]

Supplementary Information for

Probing optical anapoles with fast electron beams

Carlos Maciel-Escudero[1,2,*], Andrew B. Yankovich[3,*], Battulga Munkhbat[3,4], Denis G. Baranov[3,5], Rainer Hillenbrand[2,6], Eva Olsson[3], Javier Aizpurua[1,7], and Timur O. Shegai[3]

[1]Materials Physics Center, CSIC-UPV/EHU, Paseo de Manuel Lardizabal, Donostia-San Sebastián, 20018, Spain
[2]CIC NanoGUNE BRTA and Department of Electricity and Electronics, Tolosa Hiribidea, Donostia-San Sebastián, 20018, Spain
[3]Department of Physics, Chalmers University of Technology, Gothenburg, 412 96, Sweden
[4]Department of Photonics Engineering, Technical University of Denmark, Kgs. Lyngby, Copenhagen, 2800, Denmark
[5]Center for Photonics and 2D Materials, Moscow Institute of Physics and Technology, Dolgoprudny, Moscow, 141700, Russia
[6]IKERBASQUE, Basque Foundation for Science, Bilbao, 48011, Spain
[7]Donostia International Physics Center, Paseo de Manuel Lardizabal, Donostia-San Sebastián, 20018, Spain
[1]Corresponding authors: eva.olsson@chalmers.se, aizpurua@ehu.eus, timurs@chalmers.se
[*]These authors contributed equally

Table of contents

Supplementary Note 1. COMSOL Multiphysics simulations **S4**

S1.1 Details of the numerical simulations S4

S1.2 Permittivities used in the simulations S5

Supplementary Note 2. EEL spectrum for different velocities of the electron beam **S7**

Supplementary Note 3. Modes of the cylindrical nanoresonator **S8**

Supplementary Note 4. Multipole decomposition **S10**

S4.1 Electric dipole S10

S4.2 Electric quadrupole S11

S4.3 Partial scattering contributions of the multipoles moments S12

Supplementary Note 5. Nanodisk fabrication **S14**

Supplementary Note 6. Nanodisk thickness determination **S15**

S6.1 Electron energy loss spectroscopy S15

S6.2 Tilted-view STEM imaging S16

S6.3 Optical reflection spectroscopy S16

Supplementary Note 7. Chemical composition from energy dispersive X-ray spectroscopy (EDS) **S20**

Supplementary Note 8. Experimental EEL spectra **S22**

Supplementary Note 9. Analysis of anisotropy effects in anapole excitation **S23**

Supplementary Note 10. Analysis of the anapole-exciton coupled system **S25**

S10.1 Details of the incident electric field and the scattering channels S25

S10.2 Anapole-exciton coupled system within TCMT S27

S10.3 Reproducing the EEL spectra with TCMT S31

S10.4 Parameters obtained from the TCMT results S33

Supplementary Note 11. Substrate influence in anapole excitation **S35**

References for Supplementary Information S36

Supplementary Note 1. COMSOL Multiphysics simulations

S1.1 Details of the numerical simulations

To calculate the electron energy loss (EEL) probability $\Gamma(\omega)$ shown in Figs. 1, 4 and 5 of the main text, we use the classical dielectric theory [1, 2]. We assume that the induced electric field, coming from a response of the nanodisk to the incident electromagnetic field of the electron beam, acts back on the beam, thus causing an energy loss which reveals the energy and strength of the excitation induced in the nanodisk. We assume that the electron beam is traveling in the z-direction and we model it as a vertical line (nonrecoil approximation) carrying a current $I = -I_0 e^{i\omega z/v}$, where $I_0 = 1\,\mathrm{A}$, ω is the angular frequency, $v = 0.7c$ ($200\,\mathrm{keV}$) is the velocity of the electron and c is the speed of light in vacuum. Then, the EEL probability $\Gamma(\omega)$ of the electron beam to lose an energy $\hbar\omega$ is given by the following expression

$$\Gamma(\omega) = \frac{e^2}{\pi\hbar\omega I_0}\int_{-L_{\mathrm{PML}}/2}^{L_{\mathrm{PML}}/2} \mathrm{d}z\, \mathrm{Re}[E_z^{\mathrm{ind}}(x_e, y_e, z)\, e^{-i\omega z/v}], \tag{1}$$

where e is the elementary charge, L_{PML} is the length of the simulation box (equal to $12 \times R$, R the disk radius), $\mathrm{Re}[x]$ represents the real part of the complex number x, $E_z^{\mathrm{ind}}(\mathbf{r})$ is the z-component of the induced electromagnetic field in the nanodisk and $E_z^{\mathrm{ind}}(\mathbf{r})$ is evaluated along the trajectory of the electron beam $\mathbf{r}_e(t) = (x_e = b, y_e = 0, z = vt)$. To calculate $E_z^{\mathrm{ind}}(\mathbf{r})$ of the WS_2 disk, we perform the simulation for each frequency twice: one simulation considering the nanodisk with the permittivity function of WS_2 (see Supplementary Note S1.2, Supplementary Eqs. 4-5) and another simulation considering the nanodisk with permittivity $\varepsilon = 1$, preserving the same mesh in all domains. Their difference yields $E_z^{\mathrm{ind}}(\mathbf{r})$, which can be integrated along the electron beam trajectory to find $\Gamma(\omega)$ (Supplementary Eq. 1).

To obtain the electron energy loss probability of the WS_2 flake (green lines in Figs. 4e, 4f and 5e, 5j of the main text), we calculate the non-retarded bulk loss probability $\Gamma^{\mathrm{bulk}}(\omega)$ experienced by a fast electron beam traveling a distance $d = 70\,\mathrm{nm}$ in WS_2. The calculation is performed using the following expression [3]

$$\Gamma^{\mathrm{bulk}}(\omega) = \frac{e^2 d}{(2\pi)^2 v^2 \hbar\varepsilon_0}\mathrm{Im}\left[-\frac{1}{\varepsilon_{xx}}\ln\left(\frac{\varepsilon_{zz}\,\omega^2/v^2 + \varepsilon_{xx}\,q_c^2}{\varepsilon_{zz}\,\omega^2/v^2}\right)\right], \tag{2}$$

where $\mathrm{Im}[x]$ represents the imaginary part of the complex number x, $q_c = 1\,\mathrm{\AA}^{-1}$ is the maximum

perpendicular wavevector of the electrons selected by the collection aperture of the EELS spectrometer and ε_{xx}, ε_{zz} are the in- and out-of-plane permittivies of WS_2, respectively, discussed in the next subsection.

The scattering cross sections of the disks shown in Figs. 2b and c of the main text are calculated as

$$\sigma_{\mathrm{scat}}(\omega) = \frac{1}{S_0} \oint_A \mathrm{d}^2 r \, \mathbf{S}_{\mathrm{scat}}(\mathbf{r}) \cdot \hat{\mathbf{n}}, \tag{3}$$

where $S_0 = |\mathbf{E}_{\mathrm{inc}}|^2/(2Z_0)$, $|\mathbf{E}_{\mathrm{inc}}| = 1\,\mathrm{V/m}$ is the amplitude of the incident plane wave and Z_0 is the impedance of vacuum. $\mathbf{S}_{\mathrm{scat}}(\mathbf{r})$ is the time-averaged Poynting vector, $\mathrm{d}^2 r$ is the surface element with outward normal unit vector $\hat{\mathbf{n}}$ and the integration is performed along the complete surface A of a sphere that encloses the disk.

S1.2 Permittivities used in the simulations

To obtain the numerical calculations shown in Figs. 1 and 2 of the main text, we use a constant value of $\varepsilon = 18$ for the permittivity of the high-index dielectric nanodisk. For simplicity, these simulations are performed without considering any substrate whereas numerical calculations shown in Figs. 4 and 5 are performed with the nanodisk being on top of a $50\,\mathrm{nm}$ thick substrate layer with permittivity $\varepsilon_{\mathrm{SiN}} = 4.1853$.

For the simulations shown in Figs. 4 and 5, we use the following in- and out-of-plane permittivities of WS_2 [4], $\varepsilon_{xx} = \varepsilon_{yy}$ and ε_{zz}, respectively

$$\varepsilon_{xx} = \varepsilon_{yy} = \varepsilon_{\infty} + f_{\mathrm{ex}} \frac{\omega_{\mathrm{ex}}^2}{\omega_{\mathrm{ex}}^2 - \omega^2 - i\gamma_{\mathrm{ex}}\omega}, \tag{4}$$

$$\varepsilon_{zz} = 7, \tag{5}$$

where $\varepsilon_{\infty} = 18$ is the high-frequency permittivity, $f_{\mathrm{ex}} = 0.4$ is the oscillator strength, $\omega_{\mathrm{ex}} = 1.96\,\mathrm{eV}$ is the exciton frequency and $\gamma_{\mathrm{ex}} = 90\,\mathrm{meV}$ is the exciton damping constant. The in-plane component ε_{xx} (Supplementary Eq. 4) considers only the A-exciton absorption band of the WS_2.

For comparison, we plot in Supplementary Fig. 1 the dielectric function of WS_2 obtained from Ref. [5] (see Supplementary Fig. 1a) and the dielectric function of WS_2 used in our simulations (see Supplementary Fig. 1b). The labels A, B and C in Supplementary Fig. 1 indicate the resonance peaks associated with the A-, B- and C-exciton absorption bands of WS_2.

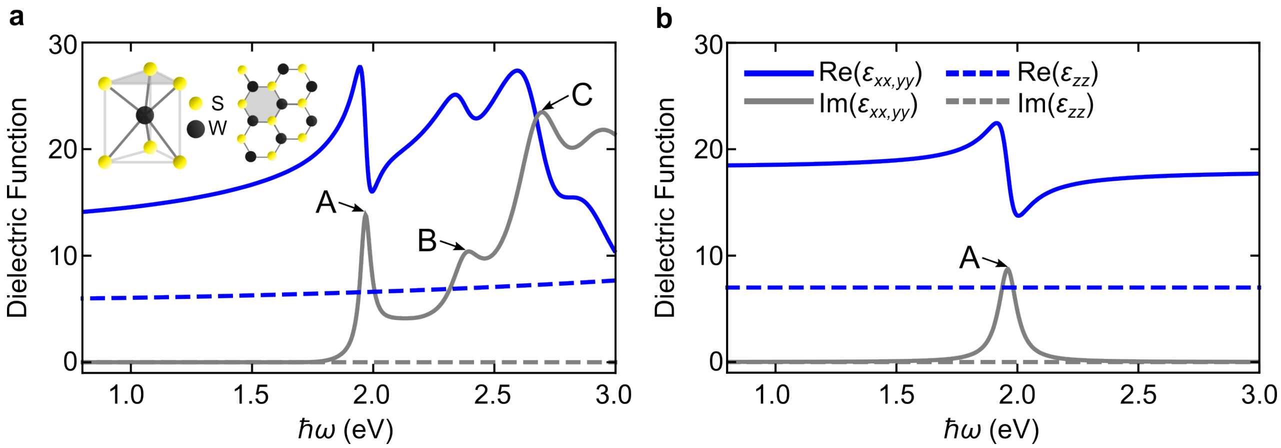

Supplementary Figure 1: **Permittivity of WS_2.** **a** Complex in- and out-of-plane permittivities of WS_2, $\varepsilon_{xx} = \varepsilon_{yy}$ (solid lines) and ε_{zz} (dashed lines), respectively. Blue lines represent real parts and gray lines imaginary parts. The dielectric functions are obtained from Ref. [5]. The labels A, B and C indicate the A-, B-, and C-exciton absorption bands of WS_2. **b** Same as **a** but the components of the dielectric function are the ones given by Supplementary Eqs. 4 and 5. Inset in panel **a** illustrates the atomic structure of WS_2.

Supplementary Note 2. EEL spectrum for different velocities of the electron beam

In Supplementary Fig. 2, we show the simulated EEL spectra of a high-index dielectric ($\varepsilon = 18$) nanodisk with radius $R = 250\,\text{nm}$ and thickness $d = 55\,\text{nm}$. We calculate the EEL spectra for the fixed impact parameter $b = 1.1R$ and different electron velocities $v = 0.8c$, $0.7c$, $0.6c$, $0.5c$ and $0.4c$. By comparing the simulated EEL spectra, we can observe that the intensity of the visible peaks diminishes as the velocity decreases. Most importantly, the peaks and dips (marked by the gray dashed lines) appear close to the same spectral positions, indicating that anapole excitation is not significantly influenced by variations in the velocity of the electron.

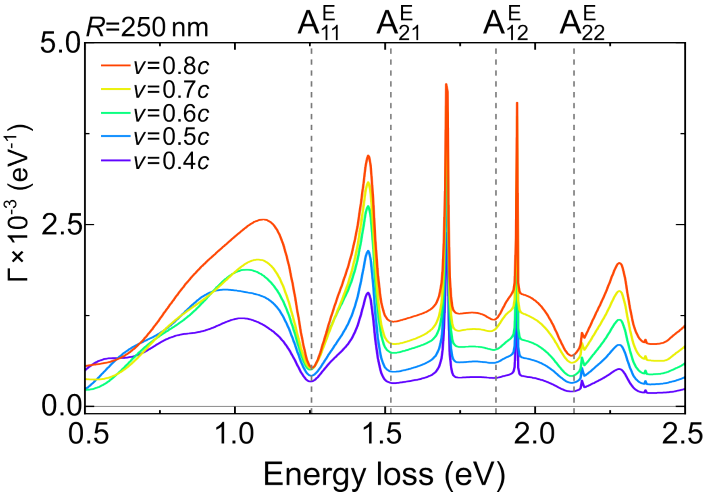

Supplementary Figure 2: **Theoretical EEL spectrum for different electron velocities v.** Comparison of simulated EEL spectra for $v = 0.8c$, $0.7c$, $0.6c$, $0.5c$ and $0.4c$, as indicated in the inset. The numerical calculations are performed for a nanodisk with $R = 250\,\text{nm}$, $d = 55\,\text{nm}$ and $\varepsilon = 18$ excited by an electron beam traveling in an aloof trajectory along the z-axis at a distance $b = 1.1R$ with respect to the nanodisk center. The gray dashed lines mark the four anapole dips A^{E}_{11}, A^{E}_{21}, A^{E}_{12} and A^{E}_{22}. Yellow spectrum is the same as the one shown in Fig. 1c of the main text.

Supplementary Note 3. Modes of the cylindrical nanoresonator

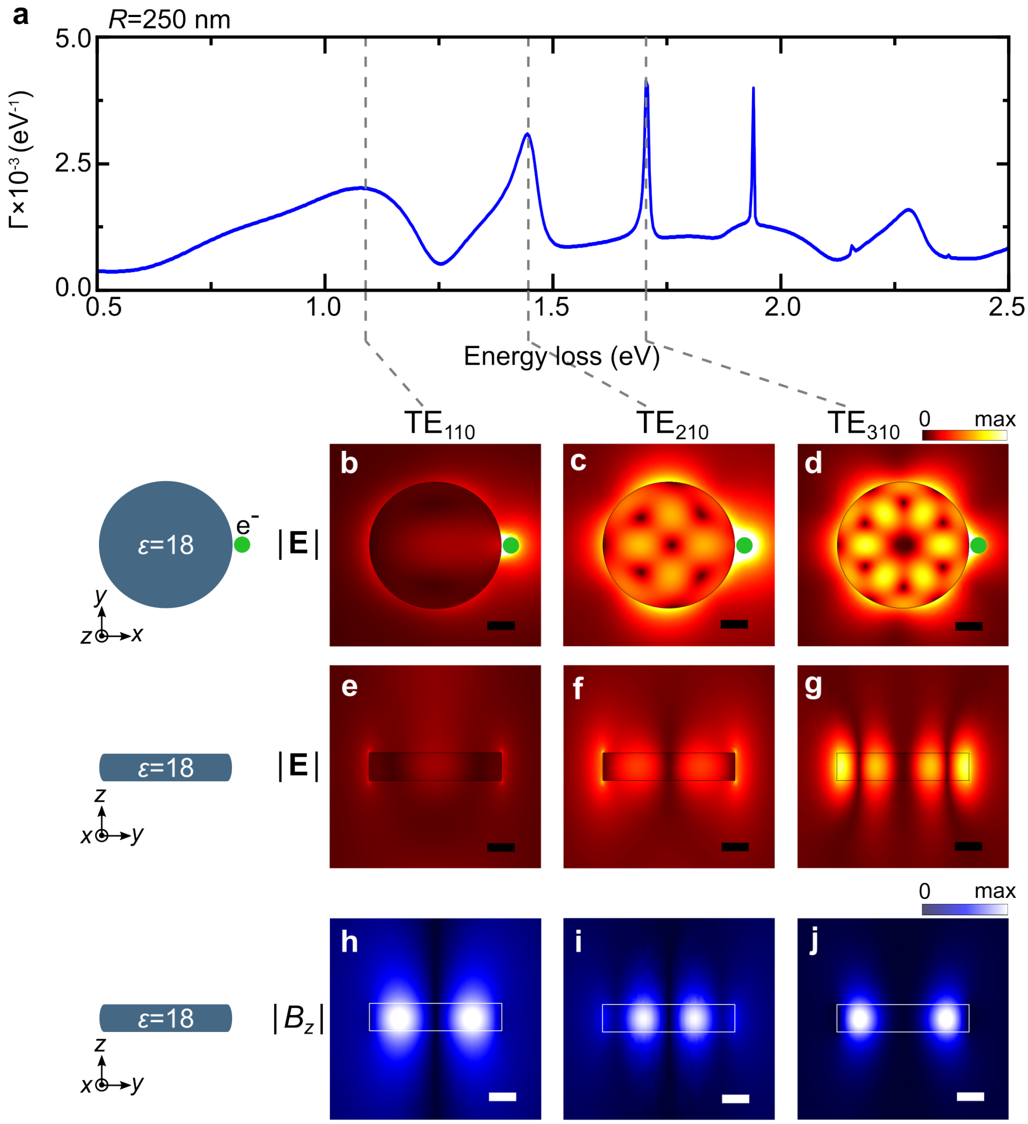

Supplementary Figure 3: **Cylindrical resonator modes. a** Simulated EEL probability $\Gamma(\omega)$ for a nanodisk with $R = 250\,\mathrm{nm}$ and $d = 55\,\mathrm{nm}$. Gray dashed lines mark the first three peak positions at $\hbar\omega_{\mathrm{TE}_{110}} = 1.09\,\mathrm{eV}$, $\hbar\omega_{\mathrm{TE}_{210}} = 1.44\,\mathrm{eV}$ and $\hbar\omega_{\mathrm{TE}_{310}} = 1.70\,\mathrm{eV}$. The field plots in panels **b-d** show the amplitude of the total electric field $|\mathbf{E}(\omega)|$ in the plane $z = 0$ (half of the nanodisk height) for the energies marked by the gray dashed lines. Green dots indicate the electron beam position. **e-g** Same as **b-d** but for the plane $x = 0$. The maximum value of $|\mathbf{E}(\omega)|$ in each case is: **b** $2 \times 10^9\,\mathrm{V/m}$, **c** $1.2 \times 10^9\,\mathrm{V/m}$, **d** $3 \times 10^9\,\mathrm{V/m}$ and **e-g** $2 \times 10^9\,\mathrm{V/m}$. **h-j** Same as **e-g** but showing the amplitude of the z-component of the total magnetic field $|B_z(\omega)|$ in the plane $x = 0$. The maximum value of $|B_z(\omega)|$ in each case is: **h** $1.7\,\mathrm{T}$, **i** $1.6\,\mathrm{T}$ and **j** $20\,\mathrm{T}$. Insets at the left of panels **b**, **e** and **h** illustrate top and side views of the nanodisk being probed by the electron beam. The scale bar in panels **b-j** is $100\,\mathrm{nm}$.

As we pointed out in the main text, one can notice that the EEL probability $\Gamma(\omega)$ spectra exhibit different peaks (see spectrum in Fig. 1c of the main text). They are attributed to the different modes

excited in the nanodisk by the fast electron beam as we discuss in the following paragraph.

In Supplementary Fig. 3a we show the $\Gamma(\omega)$ spectrum of a high-index dielectric ($\varepsilon = 18$) nanodisk of radius $R = 250\,\mathrm{nm}$ and thickness $d = 55\,\mathrm{nm}$ (same as in Fig. 1c of the main text). In the simulations, the electron beam travels with velocity $v = 0.7c$ (200 keV, c being the speed of light in vacuum) along the z-axis (symmetry axis of the nanodisk) with impact parameter $b = 1.1R$, as depicted in the inset at the left of Supplementary Fig. 3b. For simulation details, we refer the reader to the Methods section and Supplementary Note 1. We observe in Supplementary Fig. 3a clear peaks at around $1.09\,\mathrm{eV}$, $1.44\,\mathrm{eV}$, $1.70\,\mathrm{eV}$, $1.8\,\mathrm{eV}$ and $2.25\,\mathrm{eV}$. To elucidate the nature of these peaks, we show the amplitude of the total electric field $|\mathbf{E}(\omega)|$ inside the nanodisk in the plane $z = 0$ (half of the disk height) at the three energies indicated by the gray dashed lines: $1.09\,\mathrm{eV}$ (panel b), $1.44\,\mathrm{eV}$ (panel c) and $1.70\,\mathrm{eV}$ (panel d). In panel b, we observe a mode that is localized at the center and edge of the nanodisk, whereas the field plots in panels c and d reveal modes with 4 and 6 nodes along the azimuthal direction with zero nodes along the z-direction (panels e-g). Additionally, the amplitude of the z-component of the total magnetic field $|B_z(\omega)|$ is different from zero inside the disk at the energies corresponding to the peaks (see panels h-j), and thus, we classify these modes as transverse electric (TE). Furthermore, to label these modes we use the standard nomenclature of cylindrical resonator modes: $\mathrm{TE}_{n,k,p}$ where indices n, k, p denote azimuthal, radial and axial wavenumbers, respectively [6, 7].

Supplementary Note 4. Multipole decomposition

The electric spherical and Cartesian multipole moments of the nanodisks are calculated using the standard expressions reported in Refs. [8, 9]. In this section, we present the close expressions for the spherical electric dipole $\mathbf{P}_{\text{sph}}(\omega)$ and the spherical electric quadrupole $\hat{\mathbb{Q}}_{\text{sph}}(\omega)$ moments. We also show that in the long-wavelength approximation, spherical electric dipole and spherical electric quadrupole moments are a combination of Cartesian electric and toroidal multipole moments.

S4.1 Electric dipole

We calculate the spherical electric dipole moment $\mathbf{P}_{\text{sph}}(\omega)$ in the nanodisk using the following expression [8]:

$$\mathbf{P}_{\text{sph}}(\omega) = -\frac{1}{i\omega}\int_V \mathrm{d}^3r \left\{ \mathbf{J}_{\text{ind}}\, j_0(k_0r) + \frac{k_0^2}{2}\left[3(\mathbf{r}\cdot\mathbf{J}_{\text{ind}})\mathbf{r} - r^2\mathbf{J}_{\text{ind}}\right]\frac{j_2(k_0r)}{(k_0r)^2} \right\}. \tag{6}$$

Here the integral extends over the whole volume V of the nanodisk with volume element d^3r, $k_0 = \omega/c$ is the magnitude of the wavevector in vacuum, $j_\ell(x)$ is the spherical Bessel function of the first kind, r is the magnitude of the position vector $\mathbf{r} = (x, y, z)$ and $\mathbf{J}_{\text{ind}} = \mathbf{J}_{\text{ind}}(\mathbf{r})$ is the induced current density distribution in the nanodisk.

In the long-wavelength approximation ($k_0r \ll 1$), the spatial size of $\mathbf{J}_{\text{ind}}(\mathbf{r})$ is much smaller than the wavelength of the electromagnetic field, and thus one can expand the spherical Bessel functions $j_0(k_0r)$, $j_2(k_0r)$ using the small argument limit as [10]

$$j_0(k_0r) \approx 1 - \frac{(k_0r)^2}{6}, \tag{7}$$

$$j_2(k_0r) \approx \frac{(k_0r)^2}{15}. \tag{8}$$

By substituting Supplementary Eqs. 7 and 8 into Supplementary Eq. 6, we can obtain the following expression for the spherical electric dipole moment in the long-wavelength approximation

$$\begin{aligned}\mathbf{P}_{\text{sph}}^{k_0r\ll 1}(\omega) &\approx -\frac{1}{i\omega}\int_V \mathrm{d}^3r \left\{ \mathbf{J}_{\text{ind}}\left[1 - \frac{(k_0r)^2}{6}\right] + \frac{k_0^2}{2}\left[3(\mathbf{r}\cdot\mathbf{J}_{\text{ind}})\mathbf{r} - r^2\mathbf{J}_{\text{ind}}\right]\frac{(k_0r)^2}{15(k_0r)^2} \right\} \\ &\approx \underbrace{-\frac{1}{i\omega}\int_V \mathrm{d}^3r\, \mathbf{J}_{\text{ind}}}_{\mathbf{P}_{\text{car}}(\omega)} + ik_0 \underbrace{\left\{\frac{1}{10c}\int_V \mathrm{d}^3r\, \left[(\mathbf{r}\cdot\mathbf{J}_{\text{ind}})\mathbf{r} - 2r^2\mathbf{J}_{\text{ind}}\right]\right\}}_{\mathbf{T}_{\text{car}}(\omega)},\end{aligned} \tag{9}$$

where one recognizes from the last equality the conventional expressions of Cartesian electric and toroidal dipole moments [9]. From this analysis, we conclude that in the long-wavelength approximation the spherical electric dipole moment $\mathbf{P}_{\text{sph}}(\omega)$ is determined by the sum of Cartesian electric $\mathbf{P}_{\text{car}}(\omega)$ and toroidal $\mathbf{T}_{\text{car}}(\omega)$ dipole moments as:

$$\mathbf{P}_{\text{sph}}(\omega) \approx \mathbf{P}_{\text{car}}(\omega) + ik_0\mathbf{T}_{\text{car}}(\omega). \tag{10}$$

S4.2 Electric quadrupole

To calculate the spherical electric quadrupole moment $\hat{\mathbb{Q}}_{\text{sph}}(\omega)$ induced in the nanodisk, we use the following expression [8]:

$$\begin{aligned}\hat{\mathbb{Q}}_{\text{sph}}(\omega) = -\frac{3}{i\omega}\int_V \mathrm{d}^3r \Bigg\{ &\left[3(\mathbf{r}\mathbf{J}_{\text{ind}} + \mathbf{J}_{\text{ind}}\mathbf{r}) - 2(\mathbf{r}\cdot\mathbf{J}_{\text{ind}})\hat{\mathbb{I}}\right]\frac{j_1(k_0r)}{k_0r} \\ &+2k_0^2\left[5\mathbf{r}\mathbf{r}(\mathbf{r}\cdot\mathbf{J}_{\text{ind}}) - (\mathbf{r}\mathbf{J}_{\text{ind}} + \mathbf{J}_{\text{ind}}\mathbf{r})r^2 - r^2(\mathbf{r}\cdot\mathbf{J}_{\text{ind}})\hat{\mathbb{I}}\right]\frac{j_3(k_0r)}{(k_0r)^3}\Bigg\},\end{aligned} \tag{11}$$

with $\hat{\mathbb{I}}$ being the identity tensor. In the long-wavelength approximation, one can expand the spherical Bessel functions $j_1(k_0r)$, $j_3(k_0r)$ as [10]

$$j_1(k_0r) \approx \frac{k_0r}{3} - \frac{(k_0r)^3}{30}, \tag{12}$$

$$j_3(k_0r) \approx \frac{(k_0r)^3}{105}, \tag{13}$$

and substituting Supplementary Eqs. 12 and 13 into Supplementary Eq. 11 one finds that

$$\begin{aligned}\hat{\mathbb{Q}}_{\text{sph}}^{k_0r\ll 1}(\omega) &\approx -\frac{3}{i\omega}\int_V \mathrm{d}^3r \Bigg\{\left[3(\mathbf{r}\mathbf{J}_{\text{ind}} + \mathbf{J}_{\text{ind}}\mathbf{r}) - 2(\mathbf{r}\cdot\mathbf{J}_{\text{ind}})\hat{\mathbb{I}}\right]\frac{1}{k_0r}\left[\frac{k_0r}{3} - \frac{(k_0r)^3}{30}\right] \\ &\quad +2k_0^2\left[5\mathbf{r}\mathbf{r}(\mathbf{r}\cdot\mathbf{J}_{\text{ind}}) - (\mathbf{r}\mathbf{J}_{\text{ind}} + \mathbf{J}_{\text{ind}}\mathbf{r})r^2 - r^2(\mathbf{r}\cdot\mathbf{J}_{\text{ind}})\hat{\mathbb{I}}\right]\frac{(k_0r)^3}{105(k_0r)^3}\Bigg\} \\ &\approx \underbrace{-\frac{1}{i\omega}\int_V \mathrm{d}^3r\left[3(\mathbf{r}\mathbf{J}_{\text{ind}} + \mathbf{J}_{\text{ind}}\mathbf{r}) - 2(\mathbf{r}\cdot\mathbf{J}_{\text{ind}})\hat{\mathbb{I}}\right]}_{\hat{\mathbb{Q}}_{\text{car}}^{(\text{e})}(\omega)} \\ &\quad + i3k_0\underbrace{\left\{\frac{1}{42c}\int_V \mathrm{d}^3r\left[4\mathbf{r}\mathbf{r}(\mathbf{r}\cdot\mathbf{J}_{\text{ind}}) - 5r^2(\mathbf{r}\mathbf{J}_{\text{ind}} + \mathbf{J}_{\text{ind}}\mathbf{r}) + 2r^2(\mathbf{r}\cdot\mathbf{J}_{\text{ind}})\hat{\mathbb{I}}\right]\right\}}_{\hat{\mathbb{Q}}_{\text{car}}^{(\text{T})}(\omega)},\end{aligned} \tag{14}$$

where $\hat{\mathbb{Q}}^{\rm (e)}_{\rm car}(\omega)$ and $\hat{\mathbb{Q}}^{\rm (T)}_{\rm car}(\omega)$ are the conventional electric Cartesian and toroidal quadrupole moments [9], respectively. Thus, in the long-wavelength approximation spherical electric quadrupole moment $\hat{\mathbb{Q}}_{\rm sph}(\omega)$ is determined by the sum of Cartesian electric $\hat{\mathbb{Q}}^{\rm (e)}_{\rm car}(\omega)$ and toroidal $\hat{\mathbb{Q}}^{\rm (T)}_{\rm car}(\omega)$ quadrupole moments as:

$$\hat{\mathbb{Q}}_{\rm sph}(\omega) \approx \hat{\mathbb{Q}}^{\rm (e)}_{\rm car}(\omega) + i3k_0\hat{\mathbb{Q}}^{\rm (T)}_{\rm car}(\omega). \tag{15}$$

In our simulations, we calculate the multipole moments $\mathbf{P}_{\rm sph}(\omega)$, $\mathbf{P}_{\rm car}(\omega)$, $\mathbf{T}_{\rm car}(\omega)$, $\hat{\mathbb{Q}}_{\rm sph}(\omega)$, $\hat{\mathbb{Q}}^{\rm (e)}_{\rm car}(\omega)$ and $\hat{\mathbb{Q}}^{\rm (T)}_{\rm car}(\omega)$ applying the following procedure:

(i) We calculate the total electric field $\mathbf{E}(\mathbf{r})$ inside the nanodisk. The total electric field is the one produced by the probing electron plus the electric field induced in the nanodisk.

(ii) We next calculate the induced current density as $\mathbf{J}_{\rm ind}(\mathbf{r}) = -i\omega\varepsilon_0(\hat{\varepsilon}-1)\mathbf{E}(\mathbf{r})$ and perform the volume integrals (Supplementary Eqs. 6, 9, 11 and 14) with functions predefined in COMSOL Multiphysics software.

S4.3 Partial scattering contributions of the multipoles moments

From the multipole moments induced in the nanodisk, one can obtain the scattered power by the disk, $P_{\rm scat}(\omega)$, using the following expression [8, 11]:

$$\begin{aligned} P_{\rm scat}(\omega) &= P^{\mathbf{P}_{\rm sph}}_{\rm scat}(\omega) + P^{\hat{\mathbb{Q}}_{\rm sph}}_{\rm scat}(\omega) + P^{\mathbf{m}_{\rm sph}}_{\rm scat}(\omega) + \cdots, \\ &= \frac{k_0^4 c}{12\pi\varepsilon_0}\left[|\mathbf{P}_{\rm sph}(\omega)|^2 + \frac{k_0^2}{120}\left|\hat{\mathbb{Q}}_{\rm sph}(\omega)\right|^2 + \left|\frac{\mathbf{m}_{\rm sph}(\omega)}{c}\right|^2 + \cdots\right], \end{aligned} \tag{16}$$

where

$$P^{\mathbf{P}_{\rm sph}}_{\rm scat}(\omega) = \frac{k_0^4 c}{12\pi\varepsilon_0}|\mathbf{P}_{\rm sph}(\omega)|^2, \tag{17}$$

$$P^{\hat{\mathbb{Q}}_{\rm sph}}_{\rm scat}(\omega) = \frac{k_0^6 c}{1440\pi\varepsilon_0}\left|\hat{\mathbb{Q}}_{\rm sph}(\omega)\right|^2 \text{ and} \tag{18}$$

$$P^{\mathbf{m}_{\rm sph}}_{\rm scat}(\omega) = \frac{k_0^4 c}{12\pi\varepsilon_0}\left|\frac{\mathbf{m}_{\rm sph}(\omega)}{c}\right|^2, \tag{19}$$

are the scattered powers from the spherical electric dipole, spherical electric quadrupole, and spherical magnetic dipole moments, respectively. The scattered powers of higher-order electric and magnetic multipoles are implicitly indicated by the three dots in Supplementary Eq. 16. In this work,

however, we focus on the electric dipole and electric quadrupole multipole contributions.

As discussed in the previous sections, in the long-wavelength approximation, spherical electric dipole and quadrupoles can be expressed in terms of Cartesian multipoles (Supplementary Eqs. 10 and 15). Thus, to obtain the scattered power from Cartesian multipoles, we substitute Supplementary Eqs. 10 and 15 into Supplementary Eq. 16 and find that

$$P_{\text{scat}}^{k_0 r \ll 1}(\omega) \approx \frac{k_0^4 c}{12\pi\varepsilon_0} \left[\left|\mathbf{P}_{\text{car}}(\omega) + ik_0 \mathbf{T}_{\text{car}}(\omega)\right|^2 + \frac{k_0^2}{120} \left| \hat{\mathbb{Q}}_{\text{car}}^{(\text{e})}(\omega) + i3k_0 \hat{\mathbb{Q}}_{\text{car}}^{(\text{T})}(\omega) \right|^2 + \cdots \right], \quad (20)$$

where one identifies the following expressions:

$$P_{\text{scat}}^{\mathbf{P}_{\text{car}}}(\omega) = \frac{k_0^4 c}{12\pi\varepsilon_0} \left|\mathbf{P}_{\text{car}}(\omega)\right|^2, \quad (21)$$

$$P_{\text{scat}}^{\mathbf{T}_{\text{car}}}(\omega) = \frac{k_0^4 c}{12\pi\varepsilon_0} \left|ik_0 \mathbf{T}_{\text{car}}(\omega)\right|^2, \quad (22)$$

$$P_{\text{scat}}^{\hat{\mathbb{Q}}_{\text{car}}^{(\text{e})}}(\omega) = \frac{k_0^6 c}{1440\pi\varepsilon_0} \left|\hat{\mathbb{Q}}_{\text{car}}^{(\text{e})}(\omega)\right|^2 \text{ and} \quad (23)$$

$$P_{\text{scat}}^{\hat{\mathbb{Q}}_{\text{car}}^{(\text{T})}}(\omega) = \frac{k_0^6 c}{1440\pi\varepsilon_0} \left|i3k_0 \hat{\mathbb{Q}}_{\text{car}}^{(\text{T})}(\omega)\right|^2. \quad (24)$$

The partial scattering cross sections of the disk shown in Figs. 2d and 2e of the main text are calculated as

$$\begin{aligned}\sigma_{\text{scat}}(\omega) = \frac{1}{S_0} P_{\text{scat}}(\omega) &= \frac{1}{S_0} P_{\text{scat}}^{\mathbf{P}_{\text{sph}}}(\omega) + \frac{1}{S_0} P_{\text{scat}}^{\hat{\mathbb{Q}}_{\text{sph}}}(\omega) + \frac{1}{S_0} P_{\text{scat}}^{\mathbf{m}_{\text{sph}}}(\omega) + \cdots \\ &= \sigma_{\text{scat}}^{\mathbf{P}_{\text{sph}}}(\omega) + \sigma_{\text{scat}}^{\hat{\mathbb{Q}}_{\text{sph}}}(\omega) + \sigma_{\text{scat}}^{\mathbf{m}_{\text{sph}}}(\omega) + \cdots,\end{aligned} \quad (25)$$

and

$$\sigma_{\text{scat}}^{\mathbf{P}_{\text{car}}}(\omega) = \frac{1}{S_0} P_{\text{scat}}^{\mathbf{P}_{\text{car}}}(\omega), \quad (26)$$

$$\sigma_{\text{scat}}^{\mathbf{T}_{\text{car}}}(\omega) = \frac{1}{S_0} P_{\text{scat}}^{\mathbf{T}_{\text{car}}}(\omega), \quad (27)$$

$$\sigma_{\text{scat}}^{\hat{\mathbb{Q}}_{\text{car}}^{(\text{e})}}(\omega) = \frac{1}{S_0} P_{\text{scat}}^{\hat{\mathbb{Q}}_{\text{car}}^{(\text{e})}}(\omega), \quad (28)$$

$$\sigma_{\text{scat}}^{\hat{\mathbb{Q}}_{\text{car}}^{(\text{T})}}(\omega) = \frac{1}{S_0} P_{\text{scat}}^{\hat{\mathbb{Q}}_{\text{car}}^{(\text{T})}}(\omega), \quad (29)$$

where $S_0 = |\mathbf{E}_{\text{inc}}|^2/(2Z_0)$, $|\mathbf{E}_{\text{inc}}| = 1\,\text{V/m}$ is the amplitude of the incident plane wave and Z_0 is the impedance of vacuum.

Supplementary Note 5. Nanodisk fabrication

In this section we show a detailed schematics (see Supplementary Fig. 4) of the step-by-step procedure we apply to fabricate the WS_2 nanodisks on a thin SiN membrane. The details of each steps are described in Methods section of the main text.

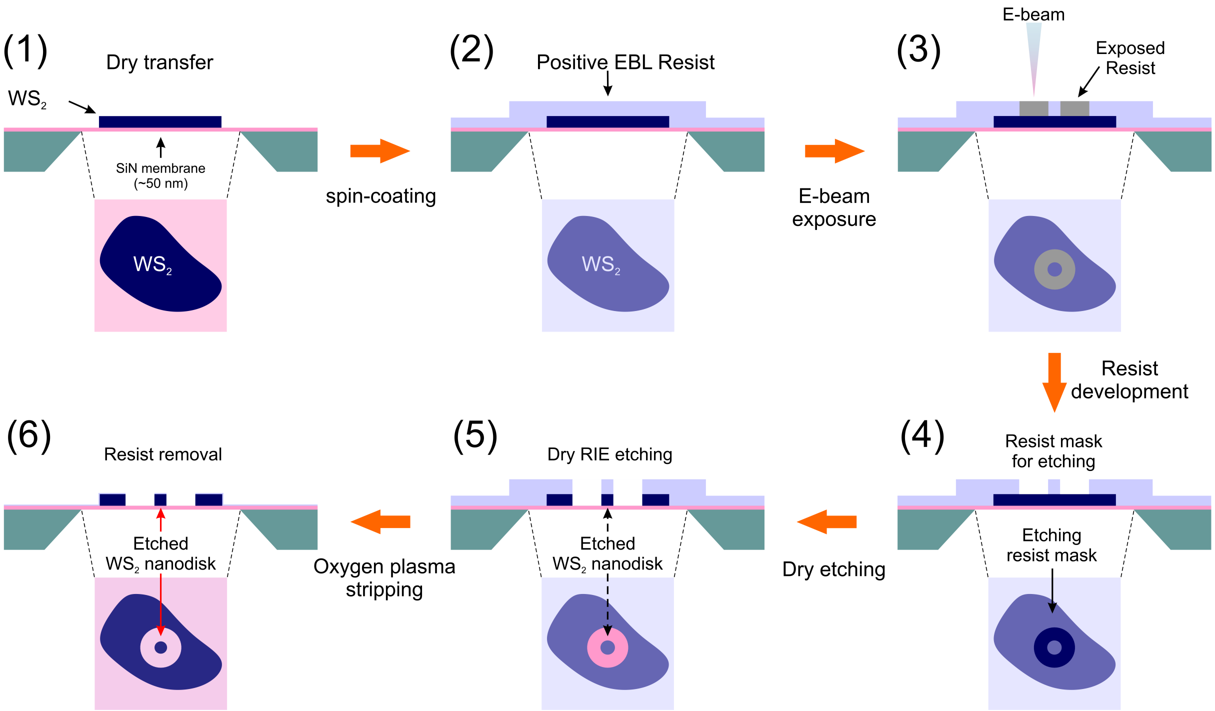

Supplementary Figure 4: **Fabrication scheme for the WS_2 nanodisks on a thin SiN TEM membrane.** It starts with (1) a dry-transfer of a mechanically-exfoliated WS_2 flake onto a $\sim 50\,\mathrm{nm}$ thick SiN membrane, and followed by (2) a spin-coating of positive an e-beam resist. In order to pattern WS_2 into various sizes of nanodisks, (3) the deposited e-beam resist was exposed using e-beam lithography with donut-shape patterns. (4) Subsequently, the exposed positive resist was developed for creating dry-etching resist mask. As a final step, we performed (5) dry-etching of the WS_2 flake through a donut-shaped resist mask, and (6) removed the left-over resist with an oxygen-plasma stripping. This process of e-beam exposure of a positive e-beam resist combined with a dry etching, enables the fabrication of donut-shaped etched patterns with isolated nanodisks at their center that have controllable diameters.

Supplementary Note 6. Nanodisk thickness determination

We determine the thickness of the WS_2 nanodisks using the following three methods: (i) electron energy loss spectroscopy, (ii) tilted-view STEM imaging and (iii) the combination of normal-incidence optical reflectivity measurements with a proper transfer-matrix fitting using a permittivity of WS_2 obtained from Ref. [5]. As discussed in the following, the thickness d extracted from each of these methods is consistent and close to the value of $70\,\mathrm{nm}$.

S6.1 Electron energy loss spectroscopy

In EELS experiments, electrons that pass through the sample are separated into a spectrum based on how much energy they lose while interacting with the sample due to inelastic scattering events. The probability that electrons undergo inelastic scattering while passing through the sample increases as the thickness of the sample is increased. As a consequence, one obtain a stronger EEL signal (associated with inelastic scattering events) when the thickness of the sample is increased. This relationship between inelastic scattering events and sample thickness allows us to measure the nanodisk thickness d by comparing the number of electrons that do not undergo inelastic scattering to the number of electrons that do. The electrons that do not undergo inelastic scattering are contained wihtin the zero-loss peak, which is typically the largest peak in an EEL spectra located at $0\,\mathrm{eV}$. The relative magnitudes of these two components are determined by the inelastic mean-free-path of the electrons in the material and thus it is straightforward to obtain the thickness of the sample in the unit of the number of inelastic mean free paths. From this value, the sample thickness can be estimated by utilizing known experimental and sample parameters with an accuracy of $\pm 10\,\%$ to $20\,\%$, as described in Ref. [12].

In practice, we estimate the thickness of the WS_2 nanodisks using this EELS method that is implemented in the Gatan Microscopy Suite 3 software (Gatan, Inc.). For our experiments, determining the nanodisk thickness using EELS is further complicated due to the SiN substrate below and residual resist material above the nanodisk. When acquiring an EEL spectrum from a nanodisk, the electron beam passes through all three layers (resist material, WS_2 and SiN) and thus the measured EEL signal is a combination of the three contributions. Disentangling the signal coming from each of these three layers is complicated without knowing the precise thickness and chemical composition of each layer. Therefore, to estimate the nanodisk thickness we first assume that the EEL signal acquired from the nanodisk only comes from WS_2, and the SiN and the residual resist contributions

are disregarded in the thickness calculation. The WS_2 nanodisk is the thickest and most strongly inelastically scattering component of the sample, and therefore should be the dominant contributor to the inelastic EEL signal. However, this assumption increases the inaccuracy of the method by an amount that is difficult to quantify. This overestimates the actual nanodisk thickness d because of the added inelastic scattering from the SiN and resist material in the EELS signal. To attempt to correct for part of this overestimation, we measure the thickness of the SiN membrane on an area of the sample where there is no WS_2 or resist material using the same EELS method, and subtract this value from the measured thickness of the nanodisk to get our final estimated nanodisk thickness. This method yields a nanodisk thickness d of $60\,\mathrm{nm} - 65\,\mathrm{nm}$. Because of the relatively large and unpredictable uncertainty associated with this methodology, we also use two other independent and more straightforward methods.

S6.2 Tilted-view STEM imaging

As a complementary method to the one discussed above, we use STEM images of tilted nanodisks to determine their thicknesses. Specifically, by acquiring a STEM image of a nanodisk that is tilted at an angle θ from the [0001] crystallographic direction of WS_2, one can measure the projected disk thickness d' defined in Supplementary Fig. 5. Once we know d', we obtain the nanodisk thickness d using the following expression:

$$d = \frac{d'}{\sin\theta}. \tag{30}$$

We acquire simultaneous SE and HAADF images of various nanodisks at a tilt angle of $\theta = 53°$ and we identify the top and bottom nanodisk edges in the images, as shown by the red ellipses in Supplementary Figs. 5a, b. The projection of the tilted circular nanodisk edges creates ellipses in the images, which can be used to determine the projected disk thickness d' (distance between the center of the top and bottom ellipse) marked by the blue line in Supplementary Figs. 5a, b. From different tilted images, we find a projected disk thickness of $d' = 57\,\mathrm{nm}$ consistently from multiple nanodisk diameters and, using Supplementary Eq. 30, we obtain a disk thickness of $d = 71\,\mathrm{nm}$. The uncertainty in this approach is limited by the accuracy of identifying the ellipse-shaped edges of the nanodisk in the images and then measuring the distance between them. We estimate the error in measuring d' to be about $5\,\mathrm{nm}$, which translates to an uncertainty in d of around $\pm 10\,\%$.

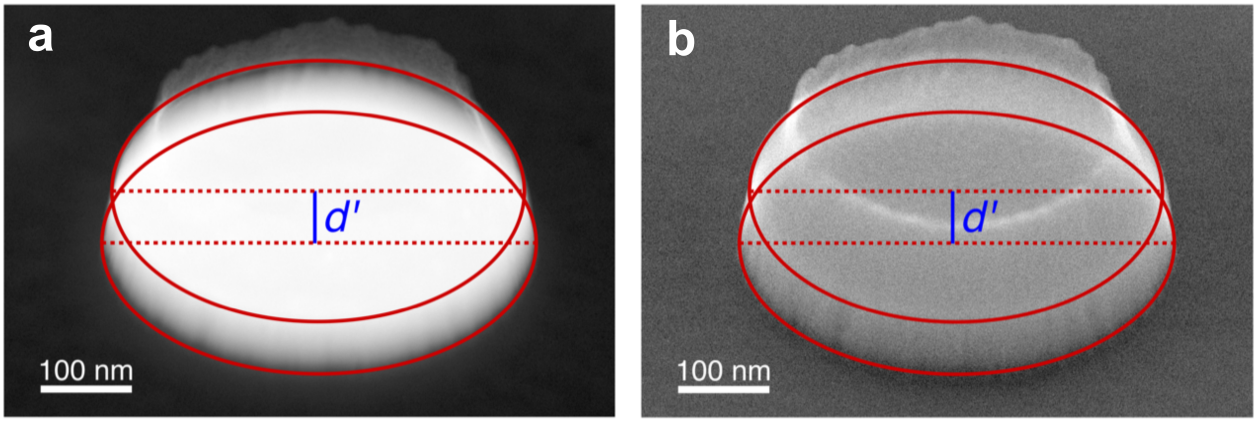

Supplementary Figure 5: **STEM imaging of a nanodisk.** Simultaneously acquired **a** HAADF and **b** SE STEM images of a nanodisk with overlays to portray how the projected disk thickness d' is determined. The red ellipses indicate the estimated edges of the top and bottom circular surfaces. The red dashed lines indicate the center line of each ellipse. The blue line indicates the distance between the center of the top and bottom ellipse, that is, d'.

S6.3 Optical reflection spectroscopy

To corroborate the nanodisk thickness obtained with EELS and STEM imaging, we additionally perform a normal-incidence optical reflectivity measurements of the WS_2 flake. Then we employ transfer-matrix simulations to find the value of the thickness d required to reproduce the position of the experimental dips in the reflectivity spectra.

Reflection spectra at normal-incidence from the WS_2 flake were collected using a $20\times$ objective (Nikon, $\mathrm{NA} = 0.45$), directed to a fiber-coupled spectrometer and normalized with reflection from a standard dielectric-coated silver mirror. The obtained reflectivity spectra are reproduced and fitted by employing the transfer-matrix simulations using the permittivity of WS_2 reported in Ref. [5]. Then we extract the thickness d of the WS_2 flake from the fitting. The transfer-matrix formalism allows to obtain the total reflection $r_{\mathrm{total}}^{s(p)}$ and transmission $t_{\mathrm{total}}^{s(p)}$ coefficients of a $s(p)$-polarized planewave, for any layered system, in terms of the thickness d_i and the dielectric function ε_i of each layer i. In our case, the nanodisk is located on top of a $50\,\mathrm{nm}$ SiN membrane (see Supplementary Note 5) and thus the multilayer system is composed of a WS_2 layer of thickness d on top of a $50\,\mathrm{nm}$ thick layer made of SiN, as depicted in Supplementary Fig. 6.

To calculate the total reflection r_{total} and reflectivity $\mathcal{R}$, we follow the transfer-matrix procedure described in Ref. [13] which we briefly discuss next. For the multilayer system shown in Supplementary Fig. 6, considering that each layer has dielectric tensor $\hat{\varepsilon}_i = \mathrm{diag}(\varepsilon_{i,x}, \varepsilon_{i,y}, \varepsilon_{i,z})$, and for normal incidence illumination, the Fresnel coefficients $r_{i,i+1}$ and $t_{i,i+1}$ of each interface between

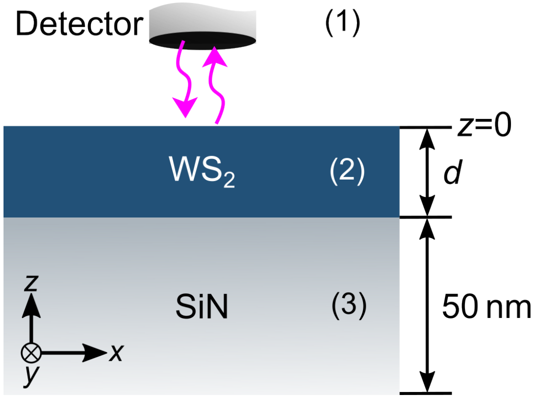

Supplementary Figure 6: **Schematics of the multilayer system.** WS_2 layer of thickness d on top of a $50\,\mathrm{nm}$ SiN layer. Labels 1, 2 and 3 refer to Air, WS_2 and SiN, respectively. The half cylinder on top of the layer system represents the detector used to collect the reflected light.

layers i and $i+1$ are given by

$$r_{i,i+1} = \frac{\sqrt{\varepsilon_{i,x}} - \sqrt{\varepsilon_{i+1,x}}}{\sqrt{\varepsilon_{i,x}} + \sqrt{\varepsilon_{i+1,x}}}, \tag{31}$$

$$t_{i,i+1} = \frac{2\sqrt{\varepsilon_{i,x}}}{\sqrt{\varepsilon_{i,x}} + \sqrt{\varepsilon_{i+1,x}}}. \tag{32}$$

We remove s and p indices from Fresnel coefficients, $r_{i,i+1}$ and $t_{i,i+1}$, since the angle of the incident illumination is zero (normal incidence). The total transfer matrix $\hat{\mathbb{T}}$ can then be obtained as

$$\hat{\mathbb{T}} = \begin{pmatrix} T_{11} & T_{12} \\ T_{21} & T_{22} \end{pmatrix} = \hat{\mathbb{M}}_{1,2}\,\hat{\mathbb{P}}_2\,\hat{\mathbb{M}}_{2,3}\,\hat{\mathbb{P}}_3\,\hat{\mathbb{M}}_{3,1}, \tag{33}$$

where each matrix $\hat{\mathbb{M}}_{i,i+1}$ and $\hat{\mathbb{P}}_i$ are determined by the following expressions

$$\hat{\mathbb{M}}_{i,i+1} = \frac{1}{t_{i,i+1}} \begin{pmatrix} 1 & r_{i,i+1} \\ r_{i,i+1} & 1 \end{pmatrix}, \tag{34}$$

$$\hat{\mathbb{P}}_i = \begin{pmatrix} e^{-ik_0 d_i \sqrt{\varepsilon_{i,x}}} & 0 \\ 0 & e^{ik_0 d_i \sqrt{\varepsilon_{i,x}}} \end{pmatrix}. \tag{35}$$

Once we obtain total matrix $\hat{\mathbb{T}}$, we use the matrix elements to calculate the reflection coefficient r_{total} and reflectivity $\mathcal{R}$ of the multilayer system as $r_{\text{total}} = \frac{T_{21}}{T_{11}}$ and $\mathcal{R} = |r_{\text{total}}|^2$.

In Supplementary Fig. 7 we show experimental reflectivity spectra (dots) together with the fits

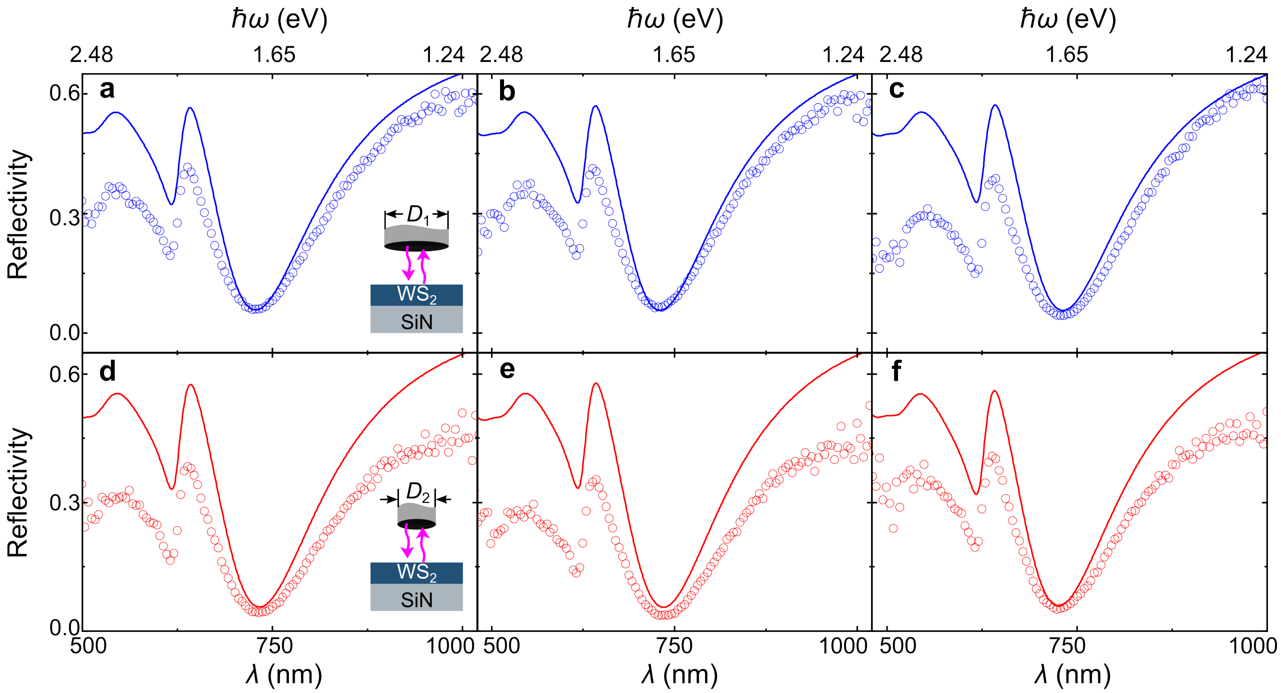

Supplementary Figure 7: **Fitting of experimental reflectivity spectra obtained from a WS_2 flake on top of the SiN membrane of 50 nm thickness.** **a**-**c** Reflectivity spectra (blue dots) collected at three different positions in the WS_2 flake. The diameter of the detection spots is $\sim 4\,\mu$m. **d**-**f** Reflectivity spectra (red dots) collected at three different positions in the WS_2 flake. The diameter of the detection spots is $\sim 2\,\mu$m. The reflectivity spectra are normalized to that obtained on a Ag reference layer of $200\,\mathrm{nm}$. In all panels, the dots are the experimental values of the reflectivity $\mathcal{R}$ and the solid lines are the simulations obtained with transfer-matrix formalism (Supplementary Eqs. 31-35). Insets in panels **a** and **d** illustrate the multilayer system and the detectors with different spot diameters $D_1 > D_2$. The fitting values obtained for the WS_2 layer thickness, d, are: **a** $74.5\,\mathrm{nm}$, **b** $74.1\,\mathrm{nm}$, **c** $74.7\,\mathrm{nm}$, **d** $75.0\,\mathrm{nm}$, **e** $75.3\,\mathrm{nm}$ and **f** $73.7\,\mathrm{nm}$.

(solid lines) obtained from the transfer-matrix formalism (Supplementary Eqs. 31-35). From the 6 fittings, we obtain an average value of $74.5\,\mathrm{nm}$ for the thickness of the WS_2, which is in good agreement with the thicknesses obtained with EELS and STEM imaging.

Supplementary Note 7. Chemical composition from energy dispersive X-ray spectroscopy (EDS)

We measure the spatial variations in chemical composition of the fabricated nanodisks using energy dispersive X-ray spectroscopy (EDS) STEM spectrum imaging. For these measurements, we raster scan a 1-2 Angstrom electron probe across the sample. For every electron probe position in the image (pixel), we collect an X-ray spectrum. This process creates a three dimensional data cube where the x and y dimensions of the data cube are the sample x and y coordinates, and the z dimension is the X-ray spectrum. We also collect the HAADF STEM image simultaneously to view the sample structure. From the EDS data cube, maps are generated which, for each pixel, integrate the EDS signal within a chosen characteristic X-ray peak. This enables the direct imaging of the nanodisk structure while simultaneously mapping the spatial variations of its constituent elements. We map the spatial variations of the W L, S K, C K, O K, F K, and Al L X-rays for three nanodisks with different diameters. All nanodisks are mapped at lower magnification to visualize the complete etched donut-shaped structure. The smallest nanodisk (with $88\,\mathrm{nm}$ radius) is also mapped both, at higher magnification to visualize smaller details around the nanodisk structure, and at a $30°$ tilt angle from the [0001] crystallographic direction of WS_2 to reveal information about the relative heights of the X-ray signals.

In Supplementary Fig. 8, we show the HAADF STEM images and extracted EDS maps of the $R = 295\,\mathrm{nm}$, $R = 110\,\mathrm{nm}$ and $R = 88\,\mathrm{nm}$ disks. We can observe that the W and the S signals come from the nanodisks at the center of the etched donut-shaped structure (see red and yellow panels). Additionally, the tungsten and sulfur X-ray signals obtained from the nanodisks are confined below the C, O, F, and Al X-ray signals (see panel e). The C, O, F, and Al X-ray signals come from the residual resist material (located above the nanodisk) that remains after the nanofabrication process (compare the first two columns in Supplementary Fig. 8e with the last four columns). From this analysis we can conclude that the residual resist material is not uniform in morphology or composition, and appears to have a tapered-edge structure and an outer cylindrical-shaped shell of different composition (higher W, O, F, and Al content) that is primarily filled with C.

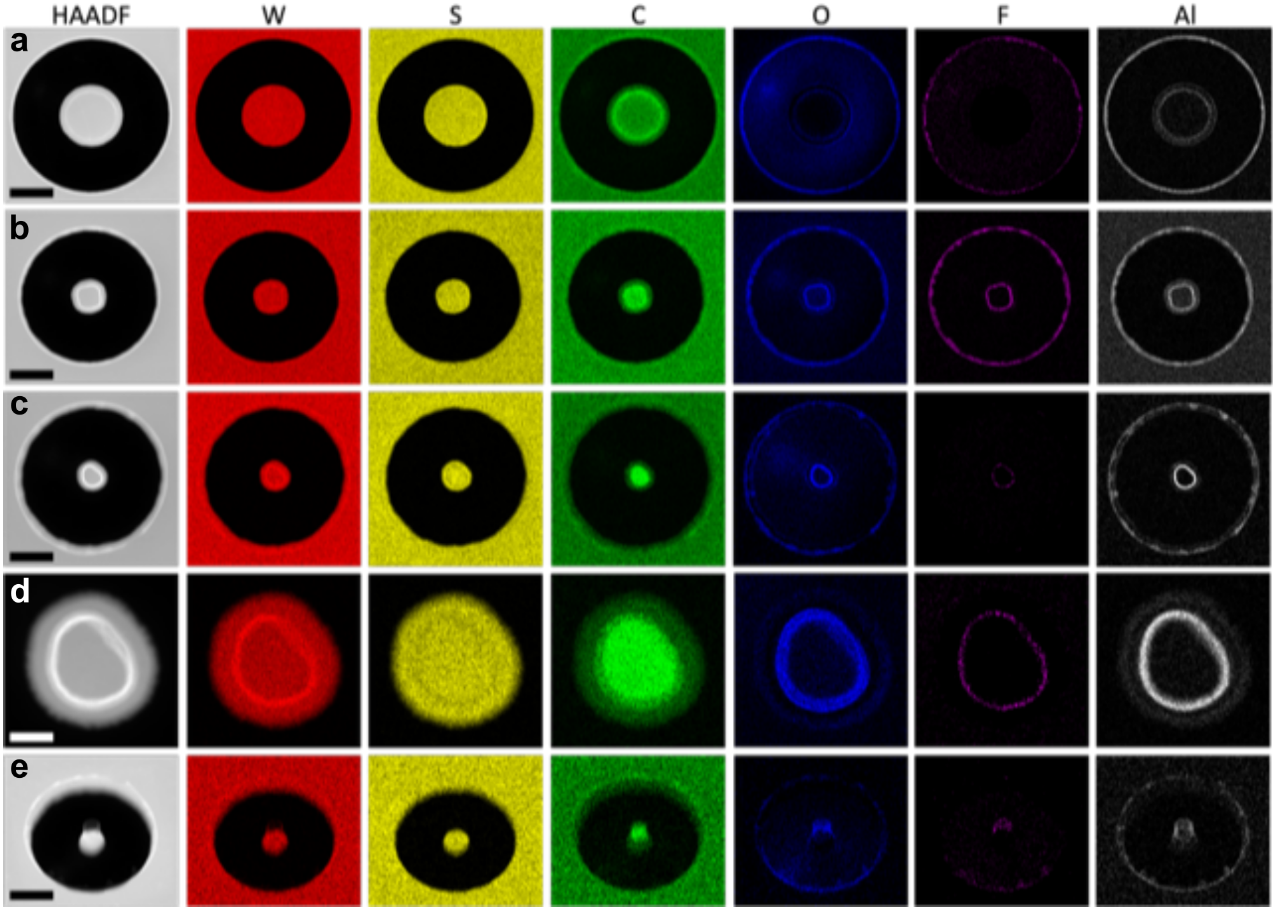

Supplementary Figure 8: **Chemical composition of the fabricated nanodisks.** STEM-EDS composition data from **a** the $R = 295\,\mathrm{nm}$ disk, **b** the $R = 110\,\mathrm{nm}$ and **c**-**e** the $R = 88\,\mathrm{nm}$ disk. **c** and **d** show a lower and higher magnification of the nanodisk, respectively. **e** Shows a 30° tilted view of the sample revealing information about the relative heights where the X-rays originate. The first column (gray) is the HAADF STEM image that is acquired simultaneously to the EDS data. The second (red), third (yellow), fourth (green), fifth (blue), sixth (magenta), and seventh (white) columns are the extracted EDS maps using the integrated signal from the W L, S K, C K, O K, F K, and Al K X-rays, respectively. The scale bars in panels **a**-**e** are $0.4\,\mu\mathrm{m}$, $0.26\,\mu\mathrm{m}$, $0.16\,\mu\mathrm{m}$, $0.04\,\mu\mathrm{m}$, and $0.2\,\mu\mathrm{m}$, respectively, and are the same for all the EDS maps in the row.

Supplementary Note 8. Experimental EEL spectra

In Supplementary Fig. 9, we show the experimental data shown in Fig. 4a of the main text.

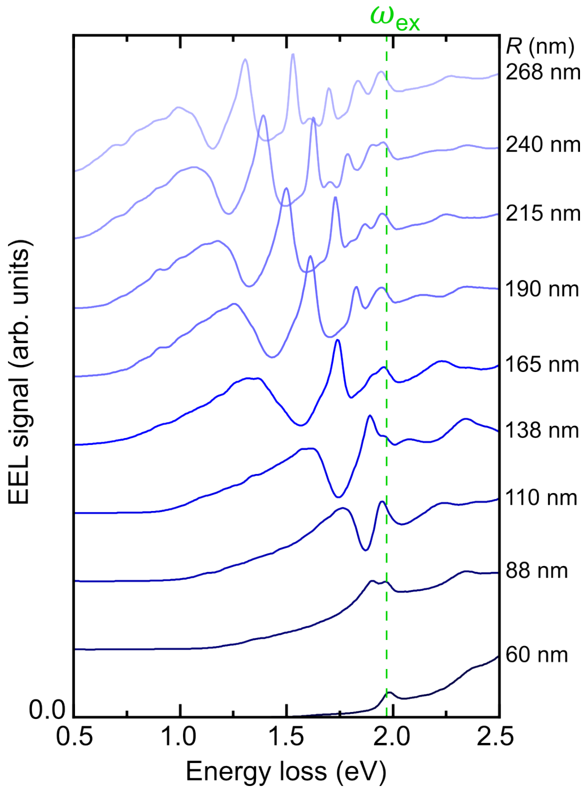

Supplementary Figure 9: **Experimental EEL spectra.** The spectra are the same as the ones shown in Fig. 4a of the main text but offset for better visualization. The green dashed line indicates the A-exciton frequency (see Supplementary Note 10).

Supplementary Note 9. Analysis of anisotropy effects in anapole excitation

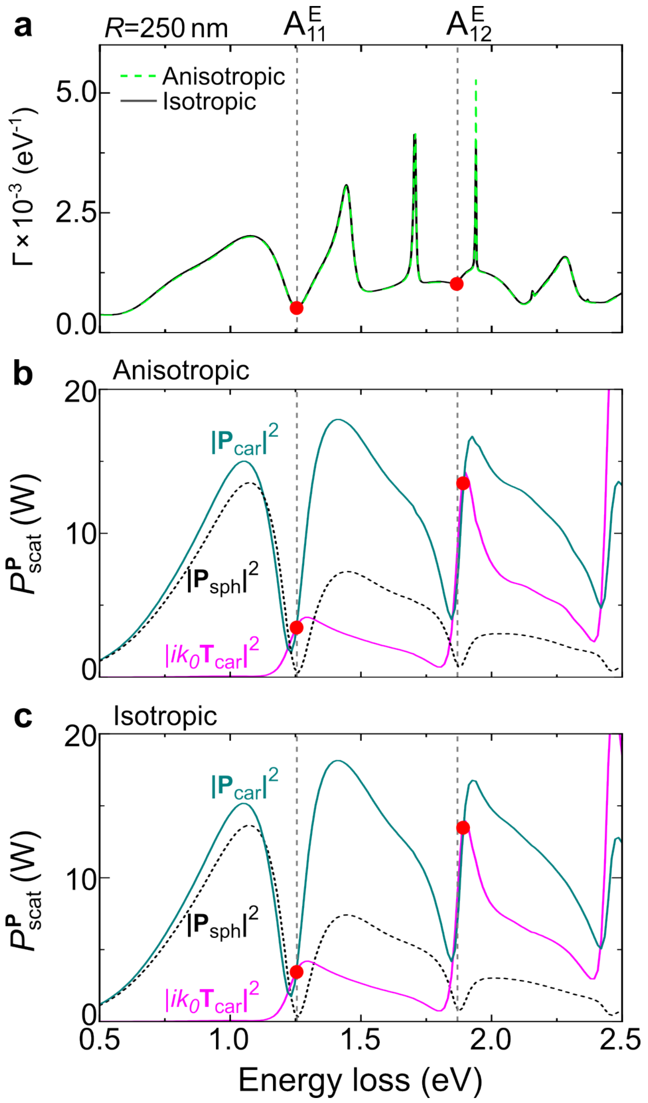

Supplementary Figure 10: **Anisotropic effects. a** Comparison of simulated EEL spectra for the anisotropic (green dashed line) and isotropic (black line) permittivity. The numerical calculations are performed for a nanodisk with radius $R = 250$ nm and thickness $d = 55$ nm excited by an electron beam traveling with velocity $v = 0.7c$ in an aloof trajectory along the z-axis at a distance $b = 1.1R$ with respect to the nanodisk center. The red dots and the gray dashed lines mark the anapole dips A^E_{11} and A^E_{12}. **b** Scattered power of the dipole moments induced in the anisotropic nanodisk. **c** Scattered power of the dipole moments induced in the isotropic nanodisk.

In Supplementary Fig. 10a we show the simulated EEL spectra (green dashed line) for a nanodisk with permittivity $\hat{\varepsilon} = \mathrm{diag}(18, 18, 7)$ (corresponding to the permittivity of WS_2 without the A-exciton resonance). For comparison, we also show the results obtained for the model disk with

$\hat{\varepsilon} = \mathrm{diag}(18, 18, 18)$ (black line) and indicate the anapole dips with the gray dashed lines. From the EEL spectra, we can observe that the effect of using an anisotropic permittivity does not modify the spectral positions of the dips, nor does it affect the excited anapole states within the nanodisk.

To further validate this claim, we calculate the spherical electric $\mathbf{P}_{\text{sph}}(\omega)$, Cartesian electric $\mathbf{P}_{\text{car}}(\omega)$ and toroidal $\mathbf{T}_{\text{car}}(\omega)$ dipole moments induced in the anisotropic nanodisk. We plot the scattered power $P^{\mathbf{P}}_{\text{scat}}(\omega)$ from these dipoles in Supplementary Fig. 10b, where one can see that the dips at around 1.25 eV and 1.87 eV are the result of the Cartesian electric dipole and toroidal dipole moments having similar amplitude but opposite phase (green and magenta lines intersect at an energies marked with $\mathrm{A}^{\mathrm{E}}_{11}$ and $\mathrm{A}^{\mathrm{E}}_{12}$ in Supplementary Fig. 10b). For comparison, we include the plot of $P^{\mathbf{P}}_{\text{scat}}(\omega)$ for the isotropic nanodisk (see Supplementary Fig. 10c) and corroborate that the intersections of the green and magenta lines coincide with the energy obtained for the anisotropic nanodisk (compare the spectral positions of the red dots in Supplementary Figs. 10b and c). Based on this analysis, we can conclude that, in our particular case, the anisotropy of WS_2 does not influence the excitation of anapole states in the nanodisk.

Supplementary Note 10. Analysis of the anapole-exciton coupled system

In order to analyse the experimental and simulated EEL spectra of the WS_2 nanodisks (Figs. 4a and 4d in the main text), we reproduce the spectra using temporal coupled mode theory (TCMT). This theoretical formalism has been widely used to model the scattered and absorbed power of an object when it is illuminated with a monochromatic plane wave [14–16]. We thus develop here an extended TCMT assuming that the incident illumination is the one produced by a fast electron beam. We then apply this formalism to reproduce the EEL spectra of the WS_2 nanodisks and to obtain the spectral dips that result from the coupling between the first electric dipole, $\mathrm{A}^{\mathrm{E}}_{11}$, anapole and the A-exciton resonance. We model the anapole-exciton system via three coupled modes excited in the disk by the probing electron. At the end of this supplementary note we show the results obtained with TCMT, together with the parameters extracted from it.

S10.1 Details of the incident electric field and the scattering channels

One of the key ingredients in TCMT is the expansion of the incident field over a set of appropriate scattering channels. In this subsection we thus show that the electric field, $\mathbf{E}_{\mathrm{bg}}(\mathbf{r};\omega)$, produced by a fast electron beam can be decomposed as a sum of incoming and outgoing spherical waves propagating toward and outward the electron beam, respectively. Each spherical wave defines a scattering channel as we discuss next.

We consider an electron traveling in vacuum along the z-axis, with impact parameter b and velocity v (see Fig. 1a of the main text). This fast electron produces a broadband electromagnetic field that can be expressed on the basis of vector spherical harmonics as follows [17]:

$$\mathbf{E}_{\mathrm{bg}}(\mathbf{r};\omega) = \sum_{\ell=0}^{\infty}\sum_{m=-\ell}^{\ell} a^{\mathrm{TE}}_{\ell m}(\omega)\,\mathbf{E}^{\mathrm{TE}}_{\ell m}(\mathbf{r};\omega) + a^{\mathrm{TM}}_{\ell m}(\omega)\,\mathbf{E}^{\mathrm{TM}}_{\ell m}(\mathbf{r};\omega), \tag{36}$$

where

$$a^{\mathrm{TE}}_{\ell m}(\omega) = -i\frac{ev}{\varepsilon_0}\frac{m}{\sqrt{\ell(\ell+1)}}\frac{\omega}{c^2}\phi^{\mathrm{TE}}_{\ell m}(\omega), \tag{37}$$

$$a^{\mathrm{TM}}_{\ell m}(\omega) = \frac{ev}{c\varepsilon_0}\frac{1}{\sqrt{\ell(\ell+1)}}\phi^{\mathrm{TM}}_{\ell m}(\omega), \tag{38}$$

and the overlap integrals $\phi_{\ell m}^{\mathrm{TE}}(\omega)$ and $\phi_{\ell m}^{\mathrm{TM}}(\omega)$ are determined as:

$$\phi_{\ell m}^{\mathrm{TE}}(\omega) = \frac{4\pi k_0}{v} i \int_{-\infty}^{\infty} \mathrm{d}z\, e^{i\omega z/v} h_\ell^{(1)}(k_0 r_e) Y_{\ell m}^*(\theta_e, \phi_e), \tag{39}$$

$$\phi_{\ell m}^{\mathrm{TM}}(\omega) = c_{\ell m}\, e^{-i\phi_e} \left(\frac{\partial}{\partial b} - \frac{i}{b}\frac{\partial}{\partial \phi_e} \right) \phi_{\ell m-1}^{\mathrm{TE}}(\omega) - d_{\ell m}\, e^{i\phi_e} \left(\frac{\partial}{\partial b} + \frac{i}{b}\frac{\partial}{\partial \phi_e} \right) \phi_{\ell m+1}^{\mathrm{TE}}(\omega). \tag{40}$$

Here $h_\ell^{(1)}(x)$ is the spherical Hankel function of the first kind, $Y_{\ell m}(\theta, \phi)$ is the scalar spherical harmonic, $c_{\ell m} = \sqrt{\ell(\ell+1) - m(m-1)}$, $d_{\ell m} = \sqrt{\ell(\ell+1) - m(m+1)}$ and the variables (r_e, ϕ_e, θ_e) are the spherical coordinates of the electron beam trajectory $\mathbf{r}_e(t) = (x_e = b, y_e = 0, z = vt)$. In Supplementary Eq. 36, the fields vectors $\mathbf{E}_{\ell m}^{\mathrm{TE}}(\mathbf{r};\omega)$ and $\mathbf{E}_{\ell m}^{\mathrm{TM}}(\mathbf{r};\omega)$ are defined according to the standard vector spherical harmonics relations:

$$\mathbf{E}_{\ell m}^{\mathrm{TE}}(\mathbf{r};\omega) = j_\ell(k_0 r)\mathbf{X}_{\ell m}(\theta, \phi), \tag{41}$$

$$\mathbf{E}_{\ell m}^{\mathrm{TM}}(\mathbf{r};\omega) = -\frac{i}{k_0}\nabla \times \left[j_\ell(k_0 r)\mathbf{X}_{\ell m}(\theta, \phi) \right], \tag{42}$$

$$\mathbf{X}_{\ell m}(\theta, \phi) = \frac{1}{\sqrt{\ell(\ell+1)}}\mathbf{L}\left[Y_{\ell m}(\theta, \varphi) \right], \tag{43}$$

where $\mathbf{L} = -i(\mathbf{r} \times \nabla)$ is the angular momentum operator. By expressing the spherical Bessel function $j_\ell(k_0 r)$ as

$$j_\ell(k_0 r) = \frac{1}{2}\left[h^{(1)}(k_0 r) + h^{(2)}(k_0 r) \right], \tag{44}$$

with $h_\ell^{(2)}(x)$ being the spherical Hankel function of the second kind, one finds that the fields vectors $\mathbf{E}_{\ell m}^{\mathrm{TE}}(\mathbf{r};\omega)$ and $\mathbf{E}_{\ell m}^{\mathrm{TM}}(\mathbf{r};\omega)$ (Supplementary Eqs. 41 and 42) can be written as the following equally weighted superposition of incoming (+) and outgoing (-) spherical waves:

$$\mathbf{E}_{\ell m}^{\mathrm{TE}}(\mathbf{r};\omega) = \underbrace{\frac{h^{(1)}(k_0 r)}{2}\mathbf{X}_{\ell m}(\theta, \phi)}_{\mathbf{E}_{\mathrm{TE},\ell m}^{+}} + \underbrace{\frac{h^{(2)}(k_0 r)}{2}\mathbf{X}_{\ell m}(\theta, \phi)}_{\mathbf{E}_{\mathrm{TE},\ell m}^{-}}, \tag{45}$$

$$\mathbf{E}_{\ell m}^{\mathrm{TM}}(\mathbf{r};\omega) = \underbrace{-\frac{i}{2k_0}\nabla \times \left[h_\ell^{(1)}(k_0 r)\mathbf{X}_{\ell m}(\theta, \phi) \right]}_{\mathbf{E}_{\mathrm{TM},\ell m}^{+}} \underbrace{-\frac{i}{2k_0}\nabla \times \left[h_\ell^{(2)}(k_0 r)\mathbf{X}_{\ell m}(\theta, \phi) \right]}_{\mathbf{E}_{\mathrm{TM},\ell m}^{-}}. \tag{46}$$

Substituting Supplementary Eqs. 45 and 46 into Supplementary Eq. 36, one further finds that the electric field $\mathbf{E}_{\mathrm{bg}}(\mathbf{r};\omega)$ can be described as the following incoming and outgoing waves (propagating

toward and outward the electron beam) coupled to different scattering channels:

$$\mathbf{E}_{\rm bg}(\mathbf{r};\omega) = \sum_{\ell=0}^{\infty}\sum_{m=-\ell}^{\ell} a_{\ell m}^{\rm TE}(\omega)\ \left[\mathbf{E}_{{\rm TE},\ell m}^{+}(\mathbf{r};\omega) + \mathbf{E}_{{\rm TE},\ell m}^{-}(\mathbf{r};\omega)\right] + \\ a_{\ell m}^{\rm TM}(\omega)\ \left[\mathbf{E}_{{\rm TM},\ell m}^{+}(\mathbf{r};\omega) + \mathbf{E}_{{\rm TM},\ell m}^{-}(\mathbf{r};\omega)\right]. \tag{47}$$

Here, each TE or TM polarization together with the pair $\{\ell, m\}$ defines a scattering channel that can be labeled as $\{q, \ell, m\}$ with q representing the polarization of the field. The power carried by the outgoing waves can be obtained by summing the radiated power along each scattering channel:

$$P_{\rm bg}(\omega) = \frac{1}{8ck_0^2}\sum_{\ell=0}^{\infty}\sum_{m=-\ell}^{\ell}\left(\left|a_{\ell m}^{\rm TE}(\omega)\right|^2 + \left|a_{\ell m}^{\rm TM}(\omega)\right|^2\right). \tag{48}$$

The incident field decomposed in different scattering channels provides a convenient tool to calculate the scattered power of an object interacting with a fast electron beam. A model of the optical anapole state (strong suppression of the scattered power) based on these scattering channels is discussed in the following section.

S10.2 Anapole-exciton coupled system within TCMT

We next consider the case of the nanodisk being illuminated by the fast electron beam. The scattering of the disk, coming from the response to the electromagnetic field of the electron beam, couples to the infinite set of scattering channels $\{q, \ell, m\}$ determined by the incident field, as sketched in Supplementary Fig. 11. The optical anapole characterized by complete suppression of the scattered field by the disk is well described by TCMT and particularly, it can be modeled as a single-channel scattering problem as we discuss next.

The first electric dipole, $\mathrm{A}_{11}^{\mathrm{E}}$, anapole state originates from the destructive interference of the radiation produced by the Cartesian electric and toroidal dipoles, both of which radiate in the TM, $\ell = 1$ channel [8, 18, 19]. This constrains the $\mathrm{A}_{11}^{\mathrm{E}}$ anapole to occur in three linearly-independent scattering channels: $\{\mathrm{TM}, 1, -1\}$, $\{\mathrm{TM}, 1, 0\}$ and $\{\mathrm{TM}, 1, 1\}$. The Cartesian electric and toroidal dipoles showing the fields of a linearly polarized dipole ($\mathbf{X}_{11} + i\mathbf{X}_{1-1}$) further constrain the $\mathrm{A}_{11}^{\mathrm{E}}$ anapole to the $m = \pm 1$ channels which have identical scattering coefficients. To model the $\mathrm{A}_{11}^{\mathrm{E}}$ anapole state excited by the fast electron beam, we thus assume that the scattered field of the nanodisk couples dominantly to the single scattering channel $\{\mathrm{TM}, 1, 1\}$. We then approximate the

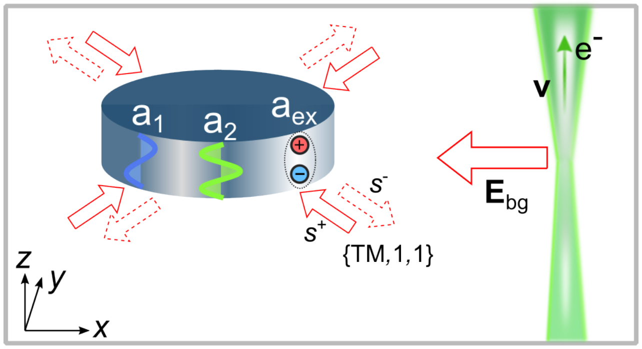

Supplementary Figure 11: **Schematics of the fast electron beam illuminating the nanodisk.** Solid arrows represent incoming waves s^+ toward the disk, whereas dashed arrows represent outgoing waves s^- outward from the disk. In the schematics, the two disk modes with amplitudes a_1, a_2 interact with the exciton with amplitude $a_{\rm ex}$. The nanodisk scatters along the channel $\{\rm TM, 1, 1\}$ to produce the $\rm A^E_{11}$ anapole state.

scattered field of the disk as follows:

$$\mathbf{E}_{\rm scat}(\mathbf{r};\omega) \approx b^{\rm TM}_{11}(\omega)\,\mathbf{E}^-_{\rm TM,11}(\mathbf{r};\omega), \tag{49}$$

with $b^{\rm TM}_{11}(\omega)$ being the amplitude of the scattered field in the $\{\rm TM, 1, 1\}$ channel. We further assume that the first electric dipole anapole is the result of the far-field coupling of two resonant modes of the disk radiating in the channel $\{\rm TM, 1, 1\}$. These resonant modes have amplitudes $a_1(t)$, $a_2(t)$, resonant frequencies ω_1, ω_2 and total decay rates γ_1, γ_2, respectively. Thus, the anapole-exciton coupled system can be modeled by considering the two resonant modes coupled to a third nonradiating mode representing the exciton resonance with amplitude $a_{\rm ex}(t)$, resonant frequency $\omega_{\rm ex}$ and absorptive decay rate $\gamma_{\rm ex}$.

Within TCMT, the dynamics of the anapole-exciton coupled system is determined by the following equation:

$$\frac{{\rm d}\mathbf{A}_3(t)}{{\rm d}t} = -i\hat{\mathbb{H}}_3\,\mathbf{A}_3(t) + \mathbf{K}_3\,s^+(t), \tag{50}$$

where $\mathbf{A}_3(t) = (a_1(t), a_2(t), a_{\rm ex}(t))^\top$ is the vector with the amplitudes of the three modes of the disk (symbol $\top$ denotes transpose of a matrix), $s^+(t)$ describes the incoming field (in our case the one produced by the electron beam) in the scattering channel $\{\rm TM, 1, 1\}$, $\mathbf{K}_3 = (\kappa_1, \kappa_2, \kappa_{\rm ex})^\top$ is the vector of radiative coupling coefficients to the incoming field, and the effective 3×3 Hamiltonian $\hat{\mathbb{H}}_3$ is given by

$$\hat{\mathbb{H}}_3 = \underbrace{\begin{pmatrix} \omega_1 & 0 & 0 \\ 0 & \omega_2 & 0 \\ 0 & 0 & \omega_{\text{ex}} \end{pmatrix}}_{\hat{\Omega}} - i \underbrace{\begin{pmatrix} \gamma_1^{\text{abs}}/2 & 0 & 0 \\ 0 & \gamma_2^{\text{abs}}/2 & 0 \\ 0 & 0 & \gamma_{\text{ex}}/2 \end{pmatrix}}_{\hat{\Gamma}_3^{\text{abs}}} - i \underbrace{\begin{pmatrix} \gamma_1^{\text{rad}}/2 & \gamma_{12} & 0 \\ \gamma_{12} & \gamma_2^{\text{rad}}/2 & 0 \\ 0 & 0 & 0 \end{pmatrix}}_{\hat{\Gamma}_3^{\text{rad}}} \tag{51}$$

$$+ \underbrace{\begin{pmatrix} 0 & 0 & g_{1\text{ex}} \\ 0 & 0 & g_{2\text{ex}} \\ g_{1\text{ex}} & g_{2\text{ex}} & 0 \end{pmatrix}}_{\hat{\mathbb{G}}_3}.$$

From Supplementary Eq. 51 one can observe that the matrix $\hat{\mathbb{H}}_3$ has the information of the resonant frequencies ω_1, ω_2, ω_{ex}, the absorptive decay rates γ_1^{abs}, γ_2^{abs}, γ_{ex} and the radiative decay rates, γ_1^{rad}, γ_2^{rad} of each mode. The total decay rate is the sum of the absorptive and radiative contributions ($\gamma = \gamma^{\text{abs}} + \gamma^{\text{rad}}$) and determines the linewidth (full width at half-maximum) of the resonance. We assume that the A_{11}^{E} anapole is the result of the far-field interference of the two resonant modes with $\gamma_1^{\text{abs}} = \gamma_1^{\text{abs}} = 0$. This far-field interference is represented by matrix elements $\gamma_{12} = \sqrt{\gamma_1^{\text{rad}}\gamma_2^{\text{rad}}/4}$ [20]. In Supplementary Eq. 51 the parameters $g_{1\text{ex}}$ and $g_{2\text{ex}}$ are the coupling strengths between each of the resonant modes and the A-exciton.

The scattered field of the disk, together with the incident field, produce the following outgoing wave

$$s^-(t) = \mathbf{D}_3^\top \mathbf{A}_3(t) + C\, s^+(t), \tag{52}$$

where $\mathbf{D}_3 = \mathbf{K}_3 = (\sqrt{\gamma_1^{\text{rad}}}, \sqrt{\gamma_2^{\text{rad}}}, 0)^\top$ and C is a constant which includes all non-resonant scattering processes in the channel [16]. In our case we assumed $C = 1$. Through a time-to-frequency Fourier transform of Supplementary Eqs. 50 and 52, we find the following steady-state solution for the amplitudes of the modes

$$\mathbf{A}_3(\omega) = \left[i(\hat{\mathbb{H}}_3 - \omega\hat{\mathbb{I}})\right]^{-1} \mathbf{D}_3\, s^+(\omega), \tag{53}$$

and the following expression for the outgoing wave

$$s^-(\omega) = \underbrace{\left[\mathbf{D}_3^\top \left[i(\hat{\mathbb{H}}_3 - \omega\hat{\mathbb{I}})\right]^{-1} \mathbf{D}_3 + C\right]}_{\hat{\mathbb{S}}(\omega)} s^+(\omega), \tag{54}$$

with $\hat{\mathbb{A}}_3(\omega) = \mathcal{F}\left[\hat{\mathbb{A}}_3(t)\right]$ and $s^{\pm}(\omega) = \mathcal{F}\left[s^{\pm}(t)\right]$. Here, $\mathcal{F}$ is the time-to-frequency Fourier transform. Equation 54 relates the incoming wave $s^{+}(\omega)$ and the outgoing wave $s^{-}(\omega)$ via the scattering matrix $\hat{\mathbb{S}}(\omega)$, which, under our assumption of a single scattering channel, reduces to a single matrix element. On the other hand, the amplitude of the outgoing wave can be written as a sum of the contributions of the scattered and the incident fields, i.e., $s^{-}(\omega) = a_{11}^{\mathrm{TM}}(\omega) + b_{11}^{\mathrm{TM}}(\omega)$. Substituting this last relation into Supplementary Eq. 54, one finds that the amplitude of the scattered field $b_{11}^{\mathrm{TM}}(\omega)$ results in

$$b_{11}^{\mathrm{TM}}(\omega) = a_{11}^{\mathrm{TM}}(\omega)\left[\hat{\mathbb{S}}(\omega) - 1\right], \tag{55}$$

where the incoming radiation is $s^{+}(\omega) = a_{11}^{\mathrm{TM}}(\omega)$.

According to Supplementary Eq. 48, the radiated power by the nanodisk can be calculated as

$$P_{\mathrm{scat}}(\omega) = |b_{11}^{\mathrm{TM}}(\omega)|^2 = |\hat{\mathbb{S}}(\omega) - 1|^2 |\, s^{+}(\omega)|^2, \tag{56}$$

whereas the absorbed power can be obtained as the sum of the absorbed power by each mode,

$$P_{\mathrm{abs}}(\omega) = \gamma_1^{\mathrm{abs}}|a_1(\omega)|^2 + \gamma_2^{\mathrm{abs}}|a_2(\omega)|^2 + \gamma_{\mathrm{ex}}|a_{\mathrm{ex}}(\omega)|^2 = 2\,\mathbf{A}_3^{\dagger}(\omega)\,\Gamma_3^{\mathrm{abs}}\,\mathbf{A}_3(\omega), \tag{57}$$

where $\hat{\mathbb{M}}^{\dagger}$ denotes conjugate transpose of matrix $\hat{\mathbb{M}}$.

Finally, to calculate the electron energy losses within the TCMT formalism, we assume that the energy lost by the electron beam when interacting with the nanodisk is equal to the energy dissipated by the nanodisk either via heating (ohmic losses) or radiation into the far field [21]. Thus, we assume that the electron energy loss probability $\Gamma(\omega)$ is proportional to the sum of the scattered and absorbed power (extinction) by the nanodisk excited by the probing electron

$$\Gamma(\omega) \propto P_{\mathrm{scat}}(\omega) + P_{\mathrm{abs}}(\omega) = |\hat{\mathbb{S}}(\omega) - 1|^2 |\, s^{+}(\omega)|^2 + 2\,\mathbf{A}_3^{\dagger}(\omega)\,\Gamma_3^{\mathrm{abs}}\,\mathbf{A}_3(\omega), \tag{58}$$

where the vector $\mathbf{A}_3(\omega)$ and the scattering matrix (a scalar in our case) $\hat{\mathbb{S}}(\omega)$ are determined by Supplementary Eqs. 53 and 54, respectively.

S10.3 Reproducing the EEL spectra with TCMT

To extract the parameters ω_1, ω_2, γ_1^{rad}, γ_2^{rad}, $g_{1\text{ex}}$ and $g_{2\text{ex}}$, we employ the developed TCMT and reproduce the EEL spectra shown in Figs. 4a and 4d of the main text. To that end, we apply the following procedure:

1. We normalize the simulated and experimental EEL spectra (Figs. 4a and 4d of the main text) to the peak maximum in the energy range between $0.5\,$eV and $2.5\,$eV. Following the description discussed in the previous section, we model the electron energy losses with Supplementary Eq. 58:
$$\Gamma(\omega) = \alpha \, \frac{P_{\text{scat}}(\omega) + P_{\text{abs}}(\omega)}{|\, s^{+}(\omega)|^2} + \beta, \tag{59}$$
where α is a proportionality constant and β is a parameter that accounts for the background in the EEL signal.

2. We reproduce using TCMT the first dip in the simulated EEL spectra of the high-index dielectric ($\varepsilon = 18$) disks with radius $R = 268\,$nm, $240\,$nm, $215\,$nm, $190\,$nm, $165\,$nm, $138\,$nm, $110\,$nm and thickness $d = 70\,$nm (see Supplementary Figs. 12a and 12d). The red lines in Supplementary Figs. 12a and 12d are guides to the eye and indicate the position of the first dip in the EEL spectra. From the TCMT results (Supplementary Fig. 12d), we obtain the eigenfrequencies ω_1, ω_2 and radiative decay rates γ_1^{rad}, γ_2^{rad} for each disk. The extracted parameters as a function of the inverse radius $1/R$ are shown in Supplementary Figs. 12g and 12h. For this step, we set $g_{1\text{ex}} = g_{2\text{ex}} = 0$ and assume that β is constant in Supplementary Eq. 59. Note in Supplementary Figs. 12g and 12h the typical linear dispersion of the Mie-resonances sustained by high-index dielectric nanostructures [22].

3. We then reproduce with TCMT the simulated EEL spectra of the WS_2 disks (Supplementary Figs. 12b and 12e) and find the coupling strengths $g_{1\text{ex}}$ and $g_{2\text{ex}}$. The red lines in Supplementary Figs. 12b and 12e are guides to the eye and indicate the position of the anapole-exciton-hybrids in the EEL spectra. In the TCMT results, the maximum values of the coupling strengths $g_{1\text{ex}}$ and $g_{2\text{ex}}$ are limited to that of the bulk polariton [13, 23] which in our case is around $0.14\,$eV. The resonance frequency and decay rate of the exciton are fixed to the values $\gamma_{\text{ex}} = 0.09\,$eV and $\omega_{\text{ex}} = 1.96\,$eV, corresponding to the same values of the WS_2 dielectric function (Supplementary Eq. 4). We limit ω_1, ω_2, γ_1^{rad} and γ_2^{rad} within a few meV from its initial value obtained in step 2. From the parameters ω_1, ω_2, ω_{ex}, γ_1^{rad}, γ_2^{rad}, γ_{ex}, $g_{1\text{ex}}$ and $g_{2\text{ex}}$, we

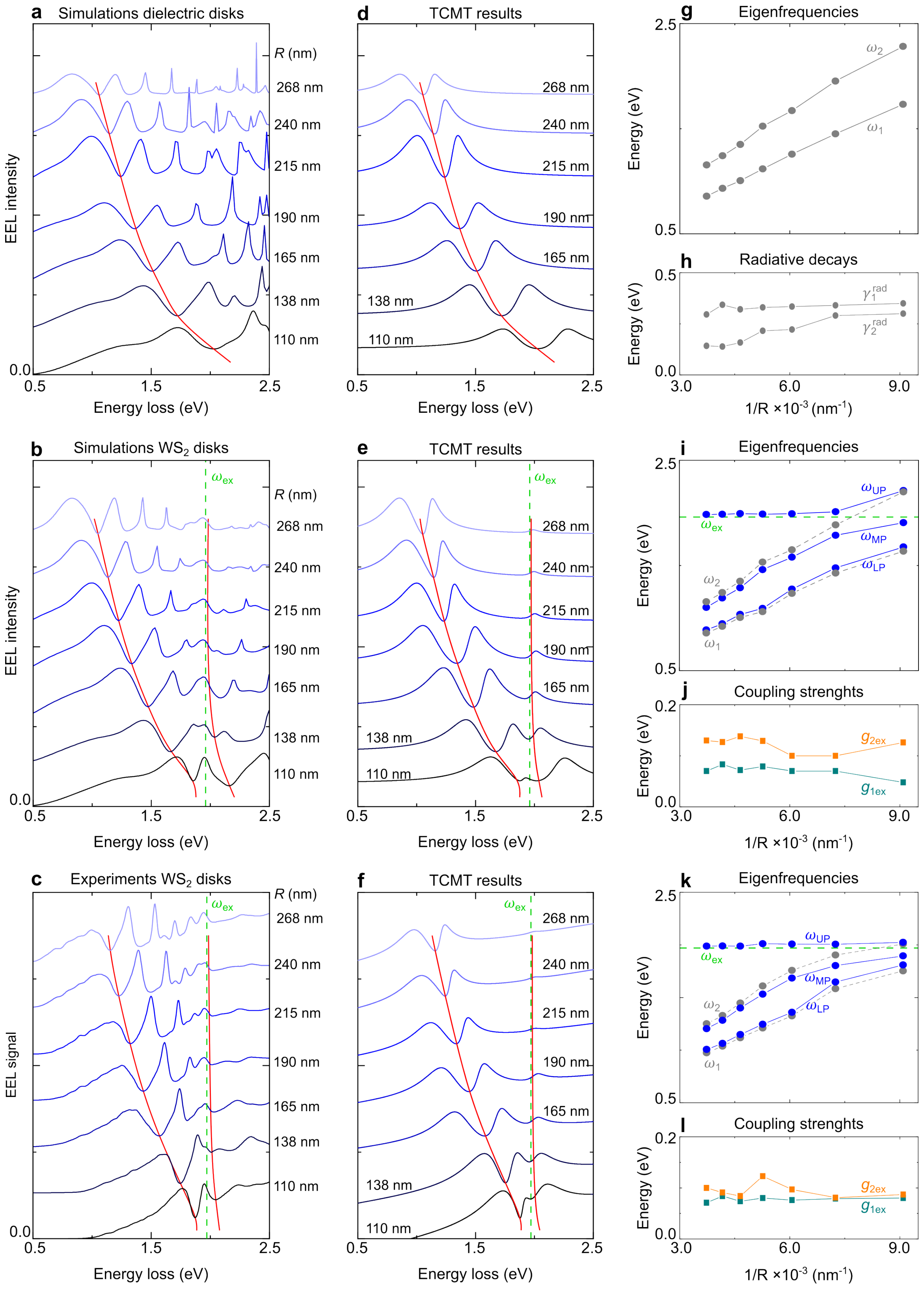

Supplementary Figure 12: **Reproducing simulated and experimental EEL spectra through TCMT.** EEL spectra obtained from **a** numerical (COMSOL) simulations of the disks with $\varepsilon = 18$, **b** COMSOL simulations of the WS_2 disks, and **c** experiments. Panels **d-f** show the EEL spectra obtained through TCMT. Panels **g-l** show the parameters extracted from the TCMT results plotted as a function of the inverse radius, $1/R$.

obtain the eigenfrequencies ω_{LP}, ω_{MP} and ω_{UP} of the hybrid modes by calculating the eigenvalues of the effective 3×3 Hamiltonian $\hat{\mathbb{H}}_3$ (Supplementary Eq. 51). The eigenfrequencies of the hybrid modes, together with the coupling strengths are shown in Supplementary Figs. 12i and 12j.

4. We repeat step 3 but for the experimental EEL spectra (Supplementary Fig. 12c). In this step, $\gamma_{\text{ex}} = 0.11\,\text{eV}$ and $\omega_{\text{ex}} = 1.97\,\text{eV}$ are fixed according to the values obtained when reproducing with TCMT the experimental EEL spectra of an unpatterned WS_2 flake (green lines shown in Figs. 4b and 4c of the main text). We assume that the background of the experimental EEL signal, β in Supplementary Eq. 59, is a linear function of energy. The TCMT results together with the extracted parameters are shown in Supplementary Figs. 12f, 12k and 12l.

S10.4 Parameters obtained from the TCMT results

Supplementary Tables 1, 2 and 3 show all parameters obtained from the TCMT results.

R (nm)	ω_1 (eV)	γ_1^{rad} (eV)	ω_2 (eV)	γ_2^{rad} (eV)
110	1.73	0.35	2.28	0.30
138	1.45	0.34	1.95	0.29
165	1.26	0.33	1.67	0.22
190	1.12	0.33	1.52	0.22
215	1.01	0.32	1.35	0.16
240	0.93	0.34	1.24	0.14
268	0.86	0.30	1.15	0.14

Supplementary Table 1: Parameters obtained by reproducing with TCMT the EEL spectra of the high-index ($\varepsilon = 18$) dielectric disks shown in Supplementary Fig. 12a. These parameters are plotted in Supplementary Figs. 12g, h.

R (nm)	ω_1 (eV)	$\gamma_1^{\rm rad}$ (eV)	$g_{1\rm ex}$ (meV)	ω_2 (eV)	$\gamma_2^{\rm rad}$ (eV)	$g_{2\rm ex}$ (meV)	$\omega_{\rm LP}$ (eV)	$\omega_{\rm MP}$ (eV)	$\omega_{\rm UP}$ (eV)
110	1.64	0.36	48	2.20	0.30	126	1.68	1.91	2.21
138	1.43	0.34	70	1.89	0.29	100	1.48	1.79	2.01
165	1.24	0.33	70	1.65	0.22	100	1.27	1.58	1.99
190	1.06	0.33	79	1.53	0.22	129	1.09	1.46	1.99
215	1.01	0.32	72	1.35	0.16	138	1.03	1.29	1.99
240	0.92	0.34	83	1.24	0.14	127	0.94	1.19	1.99
268	0.86	0.30	70	1.15	0.14	130	0.89	1.10	1.98

Table 2: Parameters obtained by reproducing with TCMT the simulated EEL spectra of the WS_2 disks shown in Fig. 4d of the main text and Supplementary Fig. 12b. These parameters are plotted in Supplementary Figs. 12i, j.

R (nm)	ω_1 (eV)	$\gamma_1^{\rm rad}$ (eV)	$g_{1\rm ex}$ (meV)	ω_2 (eV)	$\gamma_2^{\rm rad}$ (eV)	$g_{2\rm ex}$ (meV)	$\omega_{\rm LP}$ (eV)	$\omega_{\rm MP}$ (eV)	$\omega_{\rm UP}$ (eV)
110	1.75	0.36	80	2.01	0.30	87	1.81	1.90	2.02
138	1.58	0.34	79	1.90	0.29	81	1.65	1.80	2.01
165	1.33	0.33	76	1.76	0.22	97	1.36	1.68	2.01
190	1.21	0.33	80	1.61	0.22	123	1.25	1.53	2.01
215	1.12	0.32	74	1.45	0.16	84	1.15	1.40	1.99
240	1.04	0.34	84	1.33	0.14	91	1.06	1.28	1.99
268	0.97	0.30	71	1.25	0.14	100	1.01	1.20	1.99

Table 3: Parameters obtained by reproducing with TCMT the experimental EEL spectra of the WS_2 disks shown in Fig. 4a of the main text and Supplementary Fig. 12c. These parameters are plotted in Supplementary Figs. 12k, l.

Supplementary Note 11. Substrate influence in anapole excitation

In Supplementary Fig. 13a, we show a comparison between the EEL spectrum of a disk with (blue line) and without (blue shaded curve) a SiN substrate. From the spectra, we clearly see that the SiN substrate shifts the peaks and dips to lower energies (redshift) but does not alter the excitation of the anapole states, as verified in Supplementary Figs. 13b, c. The electromagnetic field at the resonant frequencies (modes) is predominantly confined within the disk (see the field plots in Supplementary Fig. 13). Notably, for higher-order modes and anapoles, the field exhibits increased localization inside the disk, leading to a reduced influence of the SiN substrate.

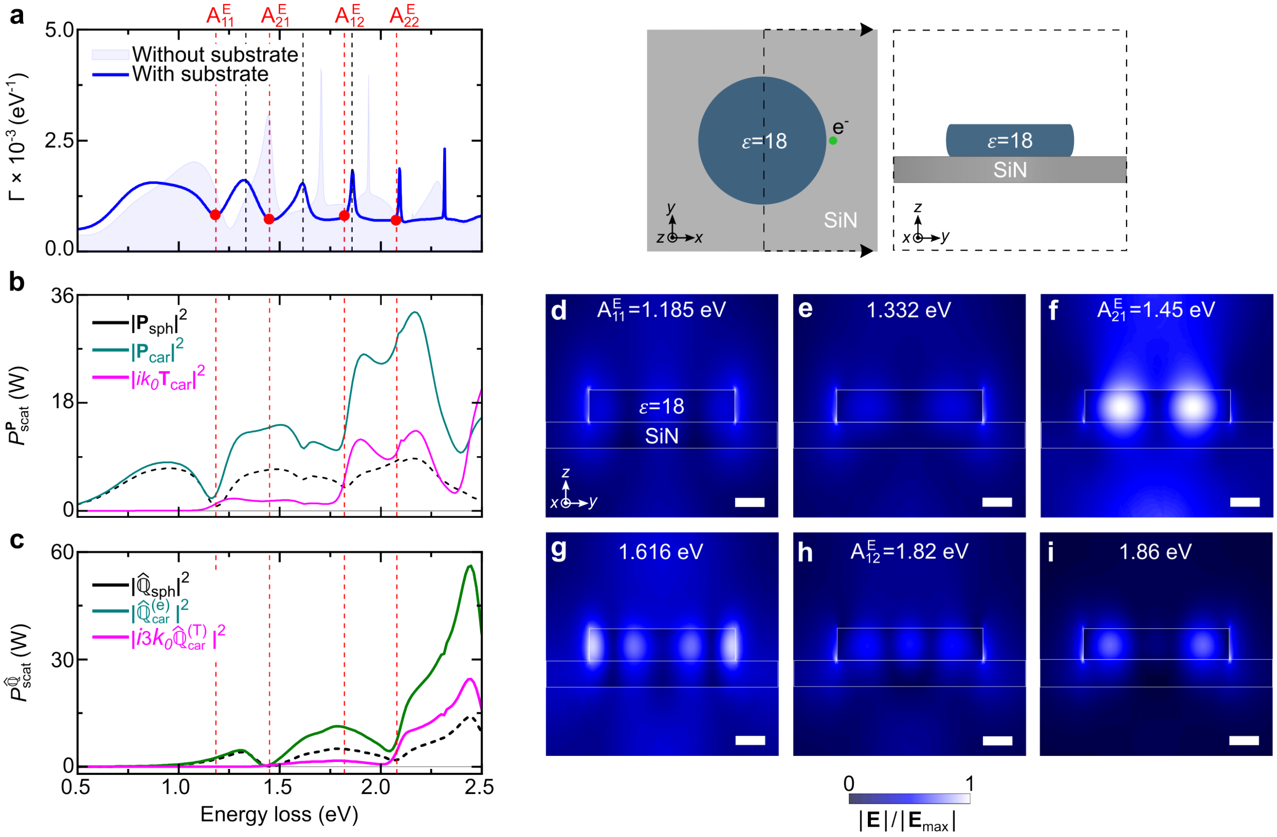

Supplementary Figure 13: **Influence of the substrate. a** Comparison of EEL spectra with (blue line) and without (blue shaded curve) a thin isotropic substrate of 50 nm thickness and constant permittivity $\varepsilon_{\mathrm{SiN}} = 4.1853$. The numerical calculations were performed for a nanodisk with radius $R = 250$ nm and thickness $d = 55$ nm excited by an electron beam traveling with velocity $v = 0.7c$ in an aloof trajectory along the z-axis at a distance $b = 1.1R$ with respect to the nanodisk center. The red dots and the red dashed lines mark the anapole dips $\mathrm{A}^{\mathrm{E}}_{11}$, $\mathrm{A}^{\mathrm{E}}_{21}$, $\mathrm{A}^{\mathrm{E}}_{12}$ and $\mathrm{A}^{\mathrm{E}}_{22}$. The black dashes lines mark the peak positions at the energies 1.332 eV, 1.616 eV and 1.86 eV. **b** Scattered power of the dipole moments in the disk on top of a substrate. **c** Scattered power of the quadrupole moments in the disk on top of a substrate. **d-i** Amplitude of the total electric field $|\mathbf{E}(\omega)|$ at the plane $x = 0$ (depicted in the schematics above the field plots) for some energies marked by the dashed lines in panel **a**. The field plots are normalized to the maximum value $|\mathbf{E}_{\mathrm{max}}|$ in each case and the scale bar is 100 nm.

References for Supplementary Information

[1] Abajo, F. J. G. D. Optical excitations in electron microscopy. *Rev. Mod. Phys.* **82**, 209–275 (2010).

[2] Hohenester, U. *Nano and Quantum Optics. An Introduction to Basic Principles and Theory* (Springer, Switzerland, 2020).

[3] Maciel-Escudero, C., Konečná, A., Hillenbrand, R. & Aizpurua, J. Probing and steering bulk and surface phonon polaritons in uniaxial materials using fast electrons: Hexagonal boron nitride. *Phys. Rev. B* **102**, 115431 (2020).

[4] Verre, R. *et al.* Transition metal dichalcogenide nanodisks as high-index dielectric mie nanoresonators. *Nat. Nanotechnol.* **14**, 679–683 (2019).

[5] Munkhbat, B., Wróbel, P., Antosiewicz, T. J. & Shegai, T. O. Optical constants of several multilayer transition metal dichalcogenides measured by spectroscopic ellipsometry in the 300–1700 nm range: High index, anisotropy, and hyperbolicity. *ACS Photonics* **9**, 2398–2407 (2022).

[6] Zhang, K. & Li, D. *Electromagnetic Theory for Microwaves and Optoelectronics* (Springer, New York, 1998), 2 edn.

[7] Bogdanov, A. A. *et al.* Bound states in the continuum and fano resonances in the strong mode coupling regime. *Adv. Photonics* **1**, 016001 (2019).

[8] Alaee, R., Rockstuhl, C. & Fernandez-Corbaton, I. An electromagnetic multipole expansion beyond the long-wavelength approximation. *Opt. Commun.* **407**, 17–21 (2018).

[9] Talebi, N., Guo, S. & van Aken, P. A. Theory and applications of toroidal moments in electrodynamics: their emergence, characteristics, and technological relevance. *Nanophotonics* **7**, 93–110 (2018).

[10] Jackson, J. D. *Classical Electrodynamics* (John Wiley & Sons, Inc, United States, 1999), third edn.

[11] Radescu, E. E. & Vaman, G. Exact calculation of the angular momentum loss, recoil force, and radiation intensity for an arbitrary source in terms of electric, magnetic, and toroid multipoles. *Phys. Rev. E* **65**, 046609 (2002).

[12] Williams, D. B. & Carter, C. B. *Transmission Electron Microscopy* (Springer, New York, 2009), 2 edn.

[13] Barra-Burillo, M. *et al.* Microcavity phonon polaritons from the weak to the ultrastrong phonon-photon coupling regime. *Nat. Commun.* **12**, 6206 (2021).

[14] Haus, H. A. *Waves and Fields in Optoelectronics* (Prentice Hall: Englewood Cliffs, New Jersey, 1984).

[15] Hamam, R. E., Karalis, A., Joannopoulos, J. D. & Soljačić, M. Coupled-mode theory for general free-space resonant scattering of waves. *Phys. Rev. A* **75**, 053801 (2007).

[16] Hsu, C. W., DeLacy, B. G., Johnson, S. G., Joannopoulos, J. D. & Soljačić, M. Theoretical criteria for scattering dark states in nanostructured particles. *Nano Lett.* **14**, 2783–2788 (2014).

[17] García de Abajo, F. J. Relativistic energy loss and induced photon emission in the interaction of a dielectric sphere with an external electron beam. *Phys. Rev. B* **59**, 3095–3107 (1999).

[18] Grahn, P., Shevchenko, A. & Kaivola, M. Electromagnetic multipole theory for optical nanomaterials. *New J. Phys.* **14**, 093033 (2012).

[19] Miroshnichenko, A. E. *et al.* Nonradiating anapole modes in dielectric nanoparticles. *Nat. Commun.* **6**, 8069 (2015).

[20] Suh, W., Wang, Z. & Fan, S. Temporal coupled-mode theory and the presence of non-orthogonal modes in lossless multimode cavities. *IEEE J. Quantum Elect.* **40**, 1511–1518 (2004).

[21] Bernasconi, G. D. *et al.* Where does energy go in electron energy loss spectroscopy of nanostructures? *ACS Photonics* **4**, 156–164 (2017).

[22] Schuller, J. A. & Brongersma, M. L. General properties of dielectric optical antennas. *Opt. Express* **17**, 24084–24095 (2009).

[23] Canales, A., Baranov, D. G., Antosiewicz, T. J. & Shegai, T. Abundance of cavity-free polaritonic states in resonant materials and nanostructures. *J. Chem. Phys.* **154**, 024701 (2021).
